# 1-Aminocyclopropane-1-carboxylic acid oxidase reaction mechanism and putative post-translational activities of the ACCO protein

**DOI:** 10.1093/aobpla/plt031

**Published:** 2013-08-01

**Authors:** David R. Dilley, Zhenyong Wang, Deena K. Kadirjan-Kalbach, Fillipos Ververidis, Randolph Beaudry, Kallaithe Padmanabhan

**Affiliations:** 1 Department of Horticulture, Michigan State University, East Lansing, MI 48824, USA; 2 Ball Horticultural Company, 622 Town Road, West Chicago, IL 60185, USA; 3 Department of Plant Biology, Michigan State University, East Lansing, MI 48823, USA; 4 Department of Plant Sciences, Technological Educational Institute of Crete, Heraklion 71004, Greece; 5 Department of Biochemistry and Molecular Biology, Michigan State University, East Lansing, MI 48824, USA

**Keywords:** ACC oxidase, ascorbate free radical, ascorbic acid, bicarbonate, cyanide, cysteine protease, phosphorylation, post-translational activities, reaction mechanism, site-directed mutagenesis.

## Abstract

1-Aminocyclopropane-1-carboxylic acid (ACC) oxidase (ACCO) catalyses the final step in ethylene biosynthesis converting ACC to ethylene, cyanide, CO_2_, dehydroascorbate and water with inputs of Fe(II), ascorbate, bicarbonate (as activators) and oxygen. Cyanide activates ACCO. A ‘nest’ comprising several positively charged amino acid residues from the C-terminal α-helix 11 along with Lys158 and Arg299 are proposed as binding sites for ascorbate and bicarbonate to coordinately activate the ACCO reaction. The binding sites for ACC, bicarbonate and ascorbic acid for *Malus domestica* ACCO1 include Arg175, Arg244, Ser246, Lys158, Lys292, Arg299 and Phe300. Glutamate 297, Phe300 and Glu301 in α-helix 11 are also important for the ACCO reaction. Our proposed reaction pathway incorporates cyanide as an ACCO/Fe(II) ligand after reaction turnover. The cyanide ligand is likely displaced upon binding of ACC and ascorbate to provide a binding site for oxygen. We propose that ACCO may be involved in the ethylene signal transduction pathway not directly linked to the ACCO reaction. ACC oxidase has significant homology with *Lycopersicon esculentum* cysteine protease LeCp, which functions as a protease and as a regulator of 1-aminocyclopropane-1-carboxylic acid synthase (*Acs2*) gene expression. ACC oxidase may play a similar role in signal transduction after post-translational processing. ACC oxidase becomes inactivated by fragmentation and apparently has intrinsic protease and transpeptidase activity. ACC oxidase contains several amino acid sequence motifs for putative protein–protein interactions, phosphokinases and cysteine protease. ACC oxidase is subject to autophosphorylaton *in vitro* and promotes phosphorylation of some apple fruit proteins in a ripening-dependent manner.

## Abstract (precis)

ACC oxidase (*Malus. domestica* ACCO1) catalyzes the final step in the biosynthesis of the plant hormone ethylene. ACCO converts 1-aminocyclopropane-1-carboxylic acid(ACC) to ethylene, cyanide, carbon dioxide and water in the presence of ferrous ion, oxygen, ascorbic acid and bicarbonate. Cyanide, a product of the reaction, activates ACCO. Site-directed mutagenesis investigations revealed binding sites for ACC, bicarbonate and ascorbic acid to include; Arg175, Arg244, Ser246, Lys158, Lys292, Arg299 and Phe300. ACCO may be involved in the ethylene signal transduction pathway not directly linked to the ACCO reaction through post-translational modifications. ACCO is subject to auto-phosphorylaton *in vitro* and promotes phosphorylation of some apple fruit proteins in a ripening-dependent manner.

## Introduction

1-Aminocyclopropane-1-carboxylic acid (ACC) oxidase (ACCO) catalyses the final step in ethylene biosynthesis. ACC oxidase requires ascorbic acid (Asc), bicarbonate, ferrous ion and dioxygen for activity.
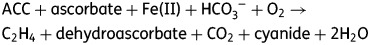



**Figure 1.**
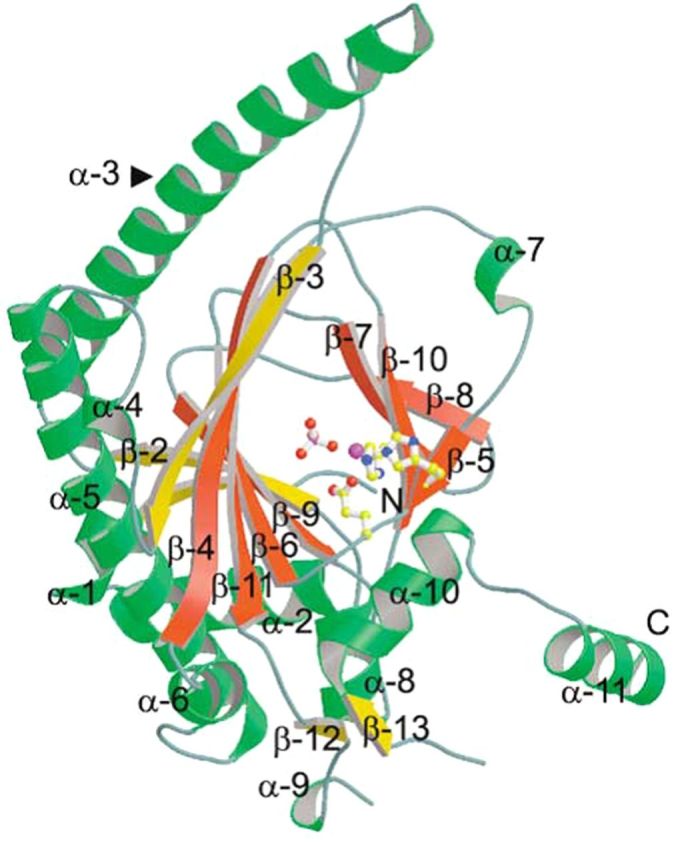
Structure of the ACCO monomer. The side chains of Fe(II) binding residues (His177, Asp179 and His234) are shown in ball and stick form. α-Helix 11 folds over the reaction site upon ACC binding (Yoo *et al.* 2006; Brisson *et al*. 2012). From: Zhang *et al.* (2004). *Chemistry and Biology* **11**:1383–1394 (with permission from *Chemistry and Biology*).

Zhang *et al.* (2004) determined the X-ray crystal structure of ACCO from petunia (*Petunia hybrida*) and found that ACCO is active as a monomer, dimer and tetramer. We have confirmed the activity of the monomer, dimer and tetramer forms of ACCO (in decreasing order of activity) by Sephadex G-200 column chromatography (data not shown). The ACCO monomer is generally reported as the active form of the enzyme. However, the results of site-directed mutagenesis and comparative modelling studies indicate that the active structure of ACCO upon ACC binding has the C-terminal α-helix 11 folded over the catalytic reaction site (Seo *et al.* 2004; Yoo *et al.* 2006; Brisson *et al.* 2012). The flexibility of Gly290 would favour the folding of α-helix 11 over the reaction centre **[see Supporting Information]**. Figure 1 shows the position of α-helix 11 away from the active site before ACC is bound. Our investigations (Kadyrzhanova *et al.* 1997, 1999; Dilley *et al.* 2001, 2003) of site-directed mutagenesis of ACCO are consistent with the ‘active structure’ as proposed.

In our investigations, native and mutant ACCO (ACCO1 from apple, *Malus domestica*) were expressed in *Escherichia coli* as His-Tag fusion proteins and purified. Steady-state enzyme kinetic experiments were performed to determine the binding sites for ACC, bicarbonate and Asc, and gain insights into the ACCO reaction mechanism. The enzyme reaction during normal turnover has an absolute dependence on Fe(II), Asc and oxygen, and requires bicarbonate for activation. His177, Asp179 and His234 are ligands for iron (Shaw *et al.* 1996; Kadyrzhanova *et al.* 1999). These His and Asp residues comprise a 2-His–1-Asp facial triad common to other mononuclear non-haem iron(II) enzymes (Hegg and Que 1997). ACC is bound to the iron centre via its amino and carboxyl groups according to spectroscopic studies with ACCO using nitric oxide (NO) as an oxygen surrogate ligand (Rocklin *et al.* 1999). Bidentate binding of ACC was confirmed by Tierney *et al.* (2005). Unless mentioned otherwise, the numbering of amino acid residues refers to the sequence of apple (*M. domestica*) ACCO1.

## Methods

### Overexpression and purification of the wild-type and mutant forms of apple ACCO1 protein

The wild-type cDNA of apple ACCO (Wilson *et al*. 1993) was cloned into the expression vector pET15b (Novagen Inc., Madison, WI, USA). The mutant forms of apple ACCO were generated by site-directed mutagenesis using the Transformer™ Site-Directed Mutagenesis Kit (Clontech Laboratories Inc., Mountain View, CA, USA**)** according to the manufacturer's instructions. The introduced point mutations were verified by manual sequencing using the Sequenase Version 2 DNA Sequencing Kit (USB Corp., Cleveland, OH, USA). The resulting gene constructs were transformed into *E. coli* strand BL21 (DE) *plysS* (Novagen Inc., Madison, WI, USA). The bacterial cells were grown at 30 °C until reaching OD_600_ = 0.3, recombinant protein expression was induced by adding 1 mM isopropyl**-**β**-**d-thiogalactoside (IPTG) and induction was continued for an additional 3 h at 30 °C before harvesting. The harvested bacterial cells were lysed by the freeze–thaw method. The lysed cells were centrifuged at 39 000 *g* for 20 min to remove cell debris. The fusion protein was purified from the supernatant (the crude extract) by using Ni^2+^-charged affinity chromatography (Novagen Inc., Madison, WI, USA) and ammonium sulfate precipitation. In general, the purification of both wild-type and mutant forms yields ∼0.5 mg of ACCO recombinant protein per litre of induced culture. The purity of the ACCO recombinant protein was confirmed by sodium dodecyl sulfate–polyacrylamide gel electrophoresis (SDS–PAGE) analysis and immunoblot analysis using ACCO monoclonal antibody. ACC oxidase activity was assayed in *E. coli* cell lysate (the crude extract) and after purification as the fusion protein. The wild-type (native) ACCO recombinant protein was always prepared in parallel and assayed along with the mutant forms of ACCO.

#### Enzyme assay

ACC oxidase assay was conducted in 5-mL stoppered vials at 20 °C for 20 min. Assays included l-ascorbate (3 mM), 20 mM NaHCO_3_, dithiothreitol (DTT) (1 mM), ACC (1 mM), 20 µM ferrous sulfate and 3-(*N*-morpholino)propanesulfonic acid (MOPS) (50 mM, pH 7.2) in 10 % glycerol in a final volume of 1 mL. The reaction was started by adding 18 or 36 µg of the purified ACCO His-Tag fusion protein or 50 µL of the crude extract (prior to Ni^2+^ affinity column purification) was added last to the assay medium. The reaction vials were shaken horizontally at 60 strokes per min. The activity of the recombinant ACCO fusion protein was similar to that of ACCO purified from apples employing the procedure of Dong *et al*. (1992). Protein concentration was determined by the Bradford procedure. Ethylene analyses were performed by injecting 1 mL of the vial headspace into a gas chromatograph employing an alumina column and a flame ionization detector. The ethylene assay readily measured 5 ppbv. Kinetic parameters were determined using PlotIT 3.2 software. The values of *K*_m_ and *K*_a_ reported in the figures and tables for ACCO mutants are apparent values although reaction velocities versus concentration of those mutants directly affecting the binding sites for substrates and activators often did not display typical Michaelis–Menten kinetics. This was most often the case when assaying mutants with bicarbonate varied where bicarbonate activation was linear rather than hyperbolic. After we discovered that ACCO inactivation was largely spared by pretreating ACCO with cyanide (0.5 mM) and α-aminoisobutyric acid (AIB) (0.5 mM), we included AIB and cyanide in enzyme assays.

### Cyanide activation studies

Preliminary studies of alternative substrates for ACCO revealed an unexpected activation and protection from inactivation when cyanide and AIB were included in an assay with ACC. Studies of cyanide activation were conducted with the ACCO His-Tag fusion protein after cutting the N-terminal His-Tag leader sequence with thrombin. The recombinant ACCO fusion protein has the sequence Gly^−4^–Ser^−3^–His^−2^–Met^−1^ on the N-terminal Met. The enzyme was desalted in 50 mM MOPS (pH 7.2) and 10 % (v/v) glycerol with a PD-10 column and stripped of iron and residual nickel on an uncharged iminodiacetate column. The enzyme was stored at −80 °C in 50 mM MOPS (pH 7.2) and 10 % glycerol. The molar extinction coefficient (*ε*) of purified ACCO at 280 nm was determined to be 2.41 × 10^4^ M^−1^ cm^−1^ and was used to calculate ACCO concentrations for the assays.

#### Enzyme assay

Enzyme activity was assayed at 20 °C in 1 mL of a reaction mixture consisting of 50 mM MOPS–HCl (pH 7.2), 3 mM l-ascorbate, 20 µM FeSO_4_, 20 mM NaHCO_3_, 1 mM DTT, 10 % (v/v) glycerol and 1 mM ACC in 5-mL stoppered vials shaken horizontally at 60 strokes per min. The assay duration was 20 min and was initiated by the addition of the enzyme at a final concentration of 1 µM unless stated otherwise. Deviations from this assay composition are stated in the figure legends. Ethylene in the vial headspace was determined by gas chromatography. Typical activity ranged from 0.7 to 1.3 nmol ethylene per nmol ACCO min^−1^ (*k*_cat/min_).

### *In vitro* phosphorylation studies

Examination of the amino acid sequence of ACCO revealed several sequence motifs for putative post-translational activities. This was not surprising given the important role that ethylene has in all phases of plant growth and development. These are listed in Table 3 and include motifs for serine/threonine kinases, tyrosine kinase, protein–protein binding and cysteine protease. To examine developmentally related kinase activity, a particulate membrane fraction of pre- and postclimacteric apple fruit proteins was incubated in [γ-^32^P]ATP in an *in vitro* phosphorylation assay for 30 min in the absence (−) or presence (+) of the ACCO fusion protein or with ACCO alone. The incubation mixtures were then passed through a Ni^2+^ affinity column with the His-Tag ACCO fusion protein attached to bind any proteins in the assay extracts capable of binding to ACCO through protein–protein interactions. The affinity column was eluted with imidazole. Proteins in the eluate (including the His-Tag ACCO) were subjected to SDS–PAGE. The gel slab was then exposed to X-ray film and stained with Coomassie Blue.

## Results

**Figure 2.**
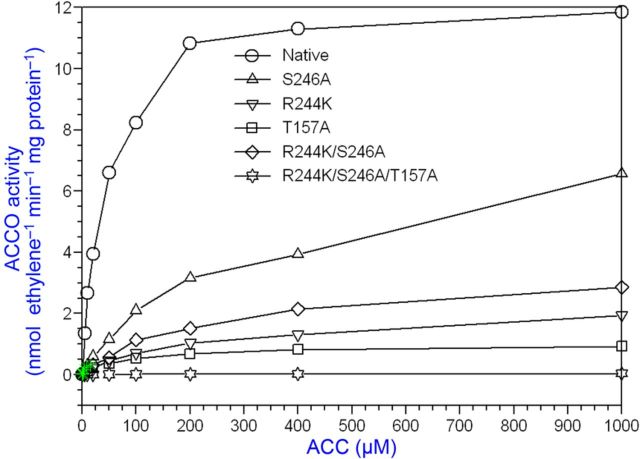
Ser246 and Arg244 are ACC binding sites in ACCO. Thr157 is essential for ACCO activity in a protein structure role. The ACC *K*_m_ is greatly elevated for the Ser246Ala and Arg244Lys mutants. The standard assay was employed using crude lysate with ACC varied. ACC oxidase activity is virtually non-detectable for the triple mutant (Ser246Ala/Arg244Lys/Thr157Ala).

**Figure 3.**
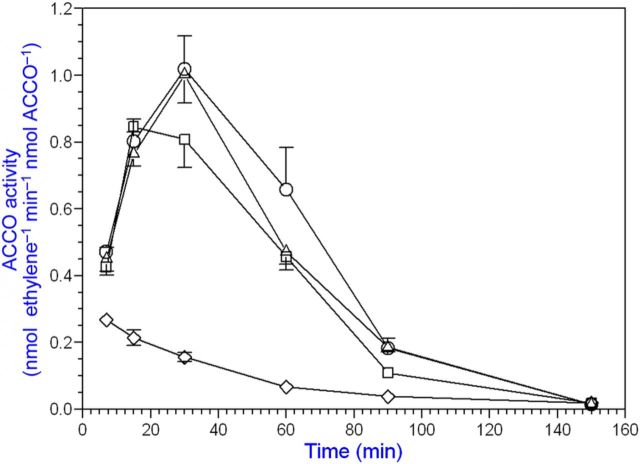
Bicarbonate pretreatment is sufficient to protect and activate ACCO. Purified recombinant ACCO was incubated with or without 20 mM bicarbonate on ice for 20 min. Then 1 nmol ACCO was assayed at 20 °C with (open circles) or without (open squares) 20 mM bicarbonate. Assay with 20 mM bicarbonate (open triangles). Assay without bicarbonate (open diamonds). Full bicarbonate activation is achieved after 30 min as a consequence of cyanide accumulation during reaction turnover.

**Figure 4.**
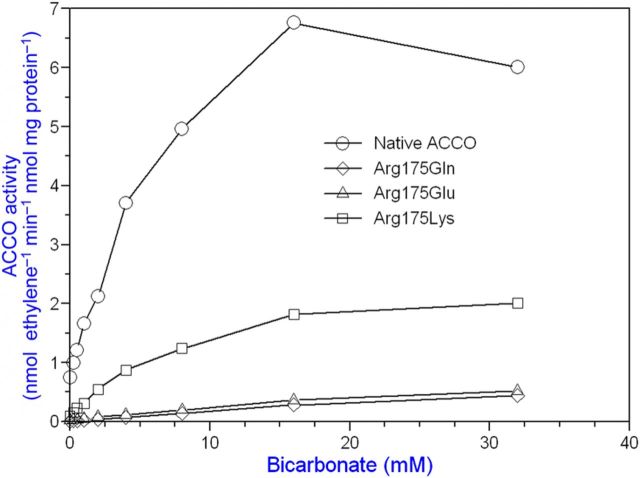
Arg175 is a critical residue for bicarbonate activation as a bicarbonate binding site or in a structural role. The low activity of the Arg175Glu and Arg175Gln mutants is linear with bicarbonate concentration, indicating a lack of a binding site. The Arg175Lys mutant with a positive charge is about one-third as active as the native ACCO. The standard assay was employed using crude lysate with bicarbonate varied.

**Figure 5.**
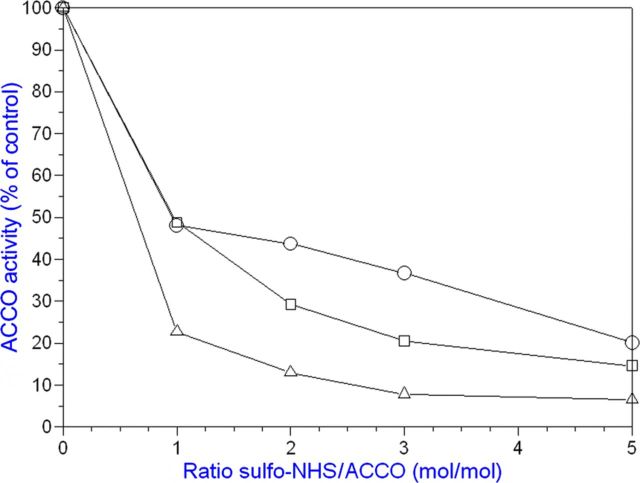
Lysine-specific reagent (*N*-hydroxysulfosuccinimide) inhibits ACCO at 1/1 and 2/1 (mol/mol) ratios. Treatment of ACCO (18.6 µmol) with reagent; circles, 2 min; squares, 5 min; and triangles, 10 min. The reaction was stopped with l-alanine (50 µM final concentration). The experiment was repeated using 10-, 15- and 20-min treatment times and all treatment times gave results similar to that of the 10-min time shown.

**Figure 6.**
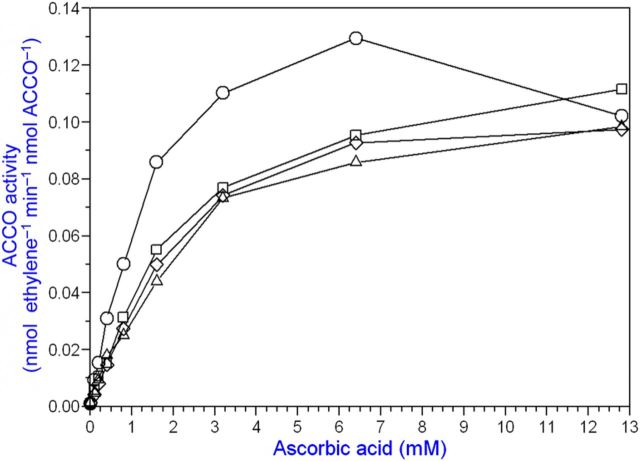
Pyridoxal 5′-phosphate is a competitive inhibitor of ACCO with respect to Asc **[see Supporting Information]**. The ACCO fusion protein was employed in the standard assay with Asc varied with four concentrations of PLP (0 mM, circles; 100 mM, squares; 200 mM, triangles; 300 mM, diamonds). Pyridoxal 5′-phosphate may form a Schiff base with the ε-amino group of lysine blocking a binding site for Asc. This is likely to be Lys292 shown to be essential for Asc activation as shown in Figure 7.

**Figure 7.**
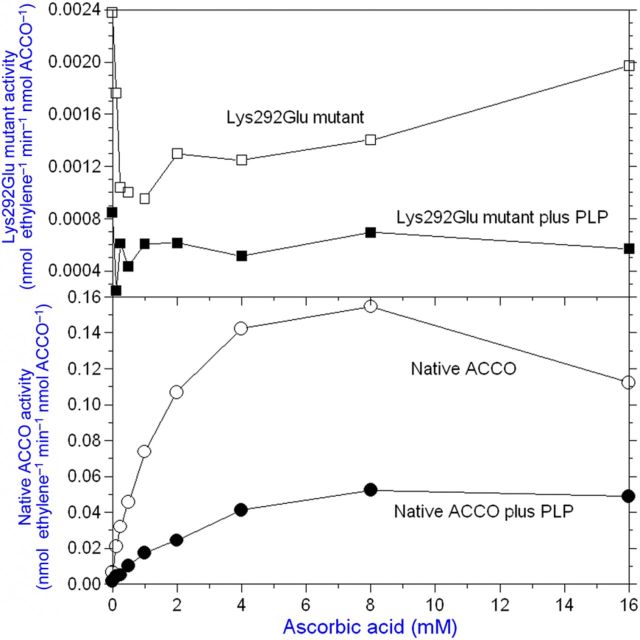
Lysine292 is an Asc binding site in ACCO (upper panel). The ACCO activity of the Lys292Glu mutant is ∼3 % that of the native ACCO and is not activated by Asc nor inhibited by PLP while the native ACCO is competitively inhibited by PLP with respect to Asc. The low residual activity of the Lys292Glu mutant is typically activated by bicarbonate (Table 1). The standard assay was employed using the ACCO fusion protein with Asc varied (lower panel).

**Figure 8.**
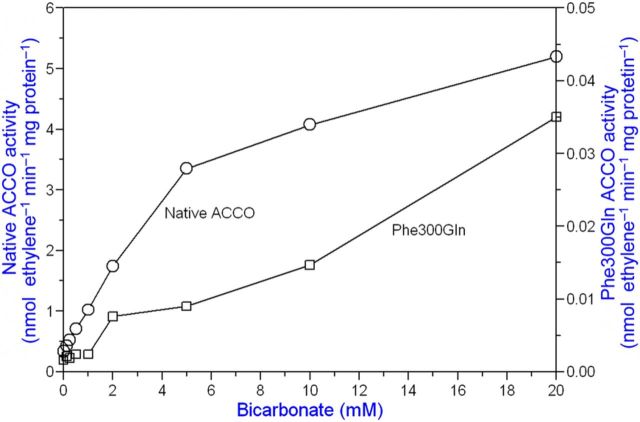
The Phe300Gln mutant is not activated by bicarbonate concentrations <2 mM. Above 2 mM bicarbonate, ACCO activity increases in a linear (non-saturable) manner, indicating that the binding site for bicarbonate is not functional. Phe300 cannot bind bicarbonate. The Phe300Gln Asc *K*_a_ = 0.52 mM and is a high-affinity binding site for Asc, indicating that Phe300 is essential for bicarbonate activation of ACCO.

**Figure 9.**
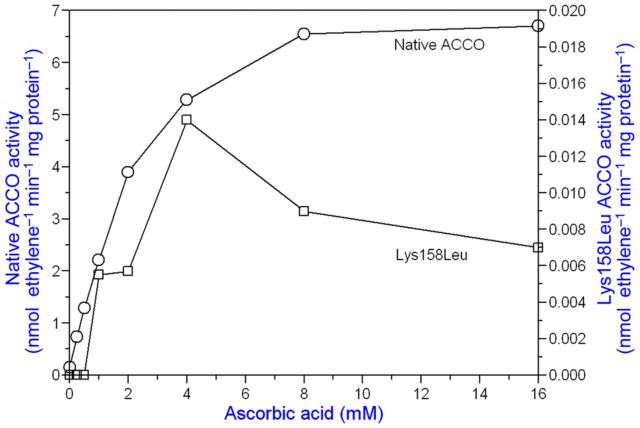
Lys158 is a high-affinity Asc binding site. The Lys158Leu mutant has very low activity and is not activated by Asc concentrations <1 mM. The crude lysate of the ACCO fusion protein was employed in the standard assay.

Bicarbonate serves as an activator and Asc as a reductant for ACCO to convert ACC to ethylene in the presence of oxygen. Our objective was to determine the binding sites for ACC, Asc and bicarbonate and their role in the reaction mechanism. The ACC substrate has been shown to bind to ACCO/Fe(II) in a bidentate manner (Rocklin *et al.* 1999). An Arg-X-Ser motif is conserved in ACCO and isopenicillin-*N*-synthase (IPNS) **[see Supporting information]**. These residues bind the carboxylate of the IPNS tripeptide substrate (Zhang *et al.* 2004) and are a common motif in 2-His–1-Asp non-haem iron enzymes (Hausinger 2004). To determine whether these Arg and Ser residues may be binding sites for ACC, we determined the Arg244Lys and Ser246Ala mutant kinetic parameters with ACC substrate varied at non-limiting Asc and bicarbonate concentrations. The ACCO Arg244Lys and Ser246Ala mutants were less active than the native enzyme and had 3- to 5-fold higher ACC *K*_m_ values, respectively, compared with that of the native enzyme (Table 1 and Fig. 2). When both Arg244 and Ser246 were mutated, activity was reduced further. Thr157 (in the β4 strand) was also mutated as Thr157Ala and examined as a double-mutant Arg244Lys/Thr157Ala and also as a triple-mutant Arg244Lys/Ser246Ala/Thr157Ala. The Thr157Ala single-point mutant did not affect the ACC *K*_m_ but drastically reduced ACCO activity by ∼14-fold. The double-mutant Arg244/Thr157 activity was reduced >1000-fold compared with that of the native enzyme. The triple-mutant (Arg244Lys/Ser246Ala/Thr157Ala) exhibited an elevated ACC *K*_m_ and lower Asc *K*_a_ and bicarbonate *K*_a_ values, 3-fold and 2-fold, respectively. The triple-mutant activity was reduced nearly 300-fold. The Arg244Lys mutant apparent *K*_a_ for Asc was not affected but the bicarbonate *K*_a_ was increased ∼4-fold. The Ser246Ala Asc *K*_a_ was reduced ∼3-fold, while the bicarbonate *K*_a_ was not affected. Arg175 was also targeted for mutation because its guanidinium group may serve as a binding site for bicarbonate. Evidence for a bicarbonate binding site in ACCO was obtained by pretreating the enzyme with or without bicarbonate and then assaying its activity with or without supplemental bicarbonate. Pretreatment with bicarbonate was sufficient to fully activate ACCO without supplemental bicarbonate (Fig. 3). Arg175 was determined to be very important for ACCO activity as seen in Fig. 4 and Table 1. The Arg175Glu mutant had a 7-fold higher ACC *K*_m_ and a normal Asc *K*_a_ but did not exhibit hyperbolic kinetics with bicarbonate (non-saturable) compared with the native enzyme. The Arg175Gln mutant exhibited a normal Asc *K*_a_ and an elevated ACC *K*_m_ and was non-saturable with bicarbonate (Fig. 4). The Arg175Lys mutant showed ACC *K*_m_ and Asc and bicarbonate *K*_a_ values similar to those of the native enzyme. The double mutants of Arg175Glu with Ser246Ala, Arg244Lys and Thr157Ala had very low activities and grossly elevated ACC *K*_m_ values and were non-saturable with bicarbonate but with near-normal Asc *K*_a_ values. Early studies of ACCO activity implicated lysine residues as potential binding sites to explain CO_2_/bicarbonate activation (Charng *et al.* 1997). ACC oxidase appears to have two essential lysine residues among its eight completely conserved Lys residues based on covalent modification studies with lysine-specific reagents AMCA-sulfo-NHS and sulfo-NHS. These sulfo-NHS reagents block the ε-amino of lysine. The lysine-specific reagent *N*-hydroxysulfosuccinimide inhibited ACCO at 1/1 and 2/1 (mol reagent/mol ACCO) ratios (Fig. 5). The experiment was repeated using 10, 15 and 20 min treatment times and all treatment times gave results similar to that of the 10 min time, confirming that ACCO has two essential lysine residues. This was also confirmed using lysine-specific reagent AMCA-sulfo-NHS which inhibited ACCO at up to a 2/1 mol/mol ratio, indicating two essential lysine residues in ACCO **[see Supporting Information]**. Pyridoxal-5′-phosphate (PLP) also reacts with the ε-amino group of lysine forming a Schiff base. We found that PLP is a competitive inhibitor of native ACCO with respect to Asc as shown in Fig. 6 **[see Supporting Information]**. The UV/Vis spectrum of ACCO in the presence of PLP was very similar to that of *N*-acetyl lysine with PLP (data not shown). The eight conserved lysine residues in ACCO are Lys144, Lys158, Lys172, Lys199, Lys230, Lys244, Lys292 and Lys296, and we constructed mutants of all of them (Tables 1 and 2). Among the lysine mutants studied, we found that the activity of Lys158Glu, Lys199Glu, Lys230Glu and Lys292Glu mutants was greatly reduced compared with that of native ACCO. Moreover, the ACC *K*_m_ and Asc and bicarbonate *K*_a_ values were differentially affected (Tables 1 and 2). When the crystal structure of ACCO was determined (Zhang *et al.* 2004), some of the conserved lysine residues (Lys158) were located close to the reaction centre, while others found to be important for enzyme activity were far removed. In particular, Lys292 in a loop between α-helix 10 and α-helix 11 (Fig. 1) when mutated to Glu greatly reduced ACCO activity. The Lys292Glu mutant had a 2-fold lower bicarbonate *K*_a_ than native ACCO and activity was reduced 16-fold (Table 1). The low activity of the Lys292Glu mutant was not activated by Asc, as seen in Fig. 7, and the low residual activity was not inhibited by PLP. The Lys292Arg mutant (a positively charged side chain) had a near-normal Asc *K*_a_ value and its activity was only reduced ∼3-fold compared with that of native ACCO. The ACC *K*_m_ and bicarbonate *K*_a_ values were not determined. This led us to mutate other amino acids in α-helix 11. As seen in Table 1, Arg299 is important for activity. The Arg299Glu mutant showed no measurable ethylene production. The Arg299Lys mutant (with a positively charged side chain) had a normal ACC *K*_m_ and typical values for Asc *K*_a_ and bicarbonate *K*_a_, and activity was ∼40 % that of native ACCO. The Arg299Leu and Arg299His mutants had normal *K*_m_ and *K*_a_ values for ACC and bicarbonate, respectively, but low Asc *K*_a_ values and very low activities. The importance of amino acids in α-helix 11 was resolved by the modelling studies of Yoo *et al*. (2006) and Brisson *et al.* (2012), indicating that α-helix 11 most likely folds over the reaction site upon ACC binding. Substrate binding in IPNS also results in the C-terminus folding over the reaction site (Zhang *et al.* 2004). Other amino acids in or near α-helix 11 are also important for ACCO activity. The activity of the Glu294Phe mutant was reduced 5-fold and that of the Glu297Leu mutant was reduced 50-fold compared with that of the native enzyme (Table 2). The Pro298Ala mutant exhibited 4-fold more activity than the native enzyme but the normal ACC *K*_m_ and *K*_a_ values for Asc and bicarbonate. Pro298 is in the C-terminus of ACCO α-helix 11 (which includes Arg299 and Phe300 binding sites for Asc and bicarbonate) and likely folds over the reaction centre upon ACC binding. Mutating Pro298 to Ala markedly increases ACCO activity without affecting the bicarbonate *K*_a_ (3.1 mM). The Pro298Ala mutant had appreciable activity when the reaction medium ingredients were scrubbed with CO_2_-free air to remove dissolved CO_2_ prior to starting the assay by adding ACCO. Similar results were obtained when ACC was varied in the standard assay, and ACC *K*_m_ values for native and mutant enzymes were closely similar (60 µM). The Asc *K*_a_ value for native ACCO was 2.67 mM, while that for the Pro298Ala mutant was 1.7 mM. Phe300Gln mutant activity was reduced 268-fold, while the ACC *K*_m_ was slightly increased and the Asc *K*_a_ decreased 4.4-fold. The Phe300Gln mutant was not activated by bicarbonate levels <1 mM and activity was not saturable with bicarbonate (Fig. 8). In contrast, Phe300Tyr mutant activity was only reduced ∼7-fold and had near-normal values for the ACC *K*_m_ and *K*_a_ values for Asc and bicarbonate (Table 2). The other α-helix 11 amino acid mutated was Glu301, and Glu301Leu mutant activity was reduced 18-fold compared with the native enzyme but showed near-normal values for the ACC *K*_m_ and *K*_a_ for Asc and bicarbonate; Glu301Asp mutant activity was only reduced ∼3-fold. Collectively, Lys292 in the loop leading to α-helix 11 along with Glu294, Glu297, Arg299, Phe300 and Glu301 in α-helix 11 are quite essential for ACCO activity. Lys158 (in β-strand 4) is also important for ACCO activity and is 7.9 Å from the iron atom (Zhang *et al.* 2004). The Lys158Arg mutant (with a positively charged side chain) had a normal ACC *K*_m_, a 4-fold lower Asc *K*_a_ and a 2-fold higher bicarbonate *K*_a_, while its activity was 14-fold lower than that of the native ACCO (Table 1). In contrast, the Lys158Gln mutant had a normal ACC *K*_m_ and Asc *K*_a_ values but activity was not saturable with bicarbonate and activity was reduced 32-fold compared with the native ACCO. The Lys158Glu mutant had a 3-fold higher ACC *K*_m_ and a 7-fold lower Asc *K*_a_ and bicarbonate activation was non-saturable while ethylene production was reduced >1000-fold. The Lys158Leu mutant had a 5-fold higher ACC *K*_m_ and a normal Asc *K*_a_ and bicarbonate activation was non-saturable while activity was 50-fold lower than that of the native ACCO. The Lys158Leu mutant was not activated by Asc concentrations <1 mM (Fig. 9). This may be related to the observation that the Phe300Gln mutant was not activated at bicarbonate concentrations <1 mM (Fig. 8). The double mutants Lys158Gln/Arg175Gln, Lys158Arg/Arg175Gln and Lys158Gln/Arg175Glu had 4- to 14-fold higher ACC *K*_m_ values and near-normal Asc *K*_a_ values and bicarbonate activation was non-saturable (Table 1). The ethylene production rates of these double mutants were reduced 23- to 300-fold compared with that of the native ACCO. As seen in Table 2, Lys230Glu mutant activity was reduced ∼17-fold, while the Asc and bicarbonate *K*_a_ values were similar to those of the native enzyme. Substituting Arg for Lys230 reduced activity only 3-fold compared with the 17-fold reduction in activity seen for the Lys230Glu mutant and lowered the bicarbonate *K*_a_ value while not changing the Asc *K*_a_ value. Lys230Gln mutant activity was reduced ∼9-fold and exhibited a 4.4-fold reduction in the bicarbonate *K*_a_. The activities of the Lys172Glu and Lys144Glu mutants were lowered slightly but the Asc *K*_a_ values were similar compared with the native ACCO. Glutamine 188 is important for ACCO activity as seen in Table 2. Substituting Asn for Gln188 (a conservative mutation) resulted in a 75-fold reduction in activity while increasing the ACC *K*_m_ value 5.6-fold but not changing the Asc or bicarbonate *K*_a_ values greatly. Glu188 is close to the active site and the site where ACCO is subject to fragmentation (during enzyme turnover) between Leu186 and Phe187 (Barlow *et al.* 1997). Mutating Phe187 to Tyr resulted in increasing ACCO activity ∼2-fold and lowered the bicarbonate *K*_a_ ∼10-fold, while not much affecting the ACC *K*_m_ or Asc *K*_a_ values. There are three conserved cysteine residues in apple ACCO: Cys28, Cys133 and Cys165. Substituting Ala for Cys in each of these positions greatly lowered enzyme activity as seen in Table 2. Among the Cys residues, Cys28 lies closest to the reaction site and its activity was reduced >100-fold with Ala in place of Cys28. The Cys133Pro mutant had about the same activity as the native enzyme. Other mutants examined were Trp203Phe, Asn216Phe and Trp251Phe and their activities were reduced 2- to 4-fold. The Trp251Phe mutant exhibited a 4-fold lower bicarbonate *K*_a_ than that of the native enzyme. The kinetic parameters of mutants listed in Tables 1 and 2 implicate Cys28, Thr157, Lys158, Arg175, Gln188, Lys199, Arg244, Ser246, Lys230, Lys292, Glu294, Glu297, Arg299, Phe300 and Glu301 as important residues affecting ACCO activity. Mutation of these amino acids differentially affects ACC *K*_m_, Asc *K*_a_ and bicarbonate *K*_a_ values. On the other hand, we found that Lys296 when mutated to Glu was more active than the native enzyme. The Pro298Ala mutant, also in α-helix 11, was ∼4-fold more active than the native ACCO and exhibited normal values for ACC *K*_m_, Asc *K*_a_ and bicarbonate *K*_a_ (Table 2).

**Table 1.** Kinetic parameters for ACCO mutants for ACC, ascorbic acid and bicarbonate binding sites. ACC oxidase activity was determined with crude lysate of the ACCO fusion protein using the standard assay. ND, not determined.

ACCO protein	ACC *K*_m_ (µM)	Asc *K*_a_ (mM)	HCO_3_ *K*_a_ (mM)	ACCO activity (nmol min^−1^ mg^−1^)
Native	51	2.3	4.9	3.48
Arg244Lys	175	2.6	17.1	1.16
Ser246Ala	277	0.88	4.7	1.74
Thr157Ala	70	1.3	0.57	0.2
Arg244Lys/Ser246Ala	175	1.4	6.1	0.92
Arg244Lys/Thr157Ala	ND	ND	ND	0.0029
Arg244Lys/Ser246ALa/Thr157Ala	245	0.77	2.4	0.01
Arg175Glu	379	2.0	51.0	0.24
Arg175Lys	83	1.5	6.3	0.65
Arg175Qln	218	2.7	21.8	0.083
Arg175Ala	193	ND	11.4	0.28
Arg175Gly	142	1.7	14.7	0.17
Arg175His	139	4.9	8.2	0.15
Arg175Glu/Ser246ALA	1200	3.6	83.0	0.061
Arg175Glu/Arg244Lys	ND	2.6	87.0	0.0024
Arg175Glu/Thr157Ala	2300	4.9	65.0	0.000064
Arg299Glu	ND	ND	ND	0.0
Arg299Lys	47	2.4	3.8	1.47
Arg299Leu	31	0.58	3.0	0.04
Arg299His	94	0.2	2.6	0.0083
Lys158Arg	46	0.6	11.0	0.25
Lys158Qln	60	2.38	34.0	0.11
Lys158Glu	173	0.32	25.0	0.0033
Lys158Leu	281	2.4	33.0	0.07
Lys158Qln/Arg175Qln	206	3.24	318.0	0.012
Lys158Arg/Arg175Qln	279	1.93	40.0	0.15
Lys158Qln/Arg175Glu	659	1.94	662.0	0.021
Lys292Glu	ND	ND	2.1	0.22
Lys292Arg	ND	1.9	ND	1.28

**Table 2.** Kinetic parameters for miscellaneous ACCO mutants influencing enzyme activity and binding sites for ACC, Asc and bicarbonate. ACC oxidase activity was determined using the ACCO fusion protein and standard assay. ND, not determined.

ACCO protein	ACC *K*_m_ (µM)	Asc *K*_a_ (mM)	HCO_3_ *K*_a_ (mM)	ACCO activity (nmol min^−1^ mg^−1^)
Native	51	2.3	4.9	3.48
Glu294Phe	ND	ND	ND	0.7
Lys296Glu	ND	ND	ND	4.16
Glu297Leu	ND	ND	ND	0.07
Pro298Ala	62	1.7	3.1	14.5
Phe300Gln	129	0.52	32.4	0.013
Phe300Tyr	36	1.0	6.2	0.47
Glu301Leu	31	1.5	5.5	0.19
Glu301Asp	ND	ND	ND	1.09
Trp203Phe	63	3.1	2.7	0.83
Asn216Phe	158	3.8	2.8	1.5
Tyr251Phe	62	1.5	1.2	1.09
Trp203Phe	63	3.1	2.7	2.0
Phe187Tyr	90	3.3	0.5	6.4
Cys28Ala	62	2.6	2.6	0.032
Cys133ALA	ND	ND	ND	0.22
Cys133Pro	ND	ND	ND	3.59
Cys165Ala	ND	ND	ND	0.13
Gln188Ala	ND	ND	ND	0.19
Gln188Asn	287	1.5	8.4	0.047
Gln188Lys	ND	ND	ND	0.12
Lys199Glu	78	0.54	3.1	0.032
Lys230Glu	ND	3.6	2.9	0.2
Lys230Qln	ND	ND	1.1	0.39
Lys230Arg	ND	3.6	1.4	1.16
Lys172Glu	ND	3.2	ND	1.75
Lys144Glu	ND	4.5	ND	2.48

### Cyanide activates and protects ACCO from inactivation

**Figure 10.**
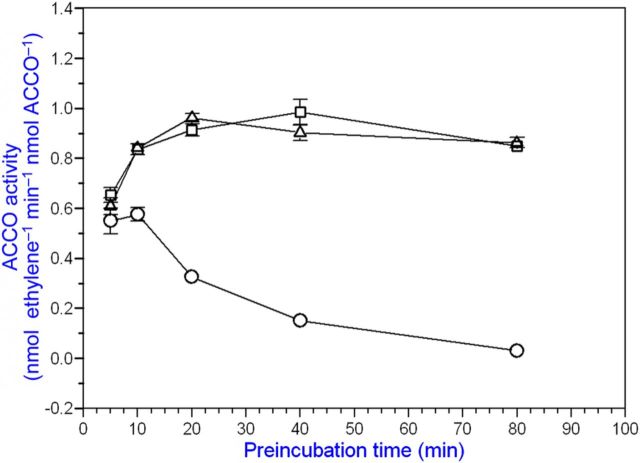
α-Aminoisobutryric acid is an alternative substrate for ACCO producing acetone, ammonia and CO_2_. In the presence of cyanide (CN^−^) and AIB, ACCO is activated and enzyme inactivation is prevented. Without CN^−^ and AIB, ACCO is inactivated. Cyanide or AIB alone only partially prevents inactivation. The ACCO fusion protein was incubated without cyanide and AIB (open circles), 250 µM KCN plus 250 µM AIB (open squares) or 500 µM KCN plus 250 µM AIB (open triangles) for up to 2 h in the standard assay without ACC. During this time, AIB was presumed to be oxidized to products. At the times indicated, aliquots of the assay mixture were assayed in the standard assay with ACC.

**Figure 11.**
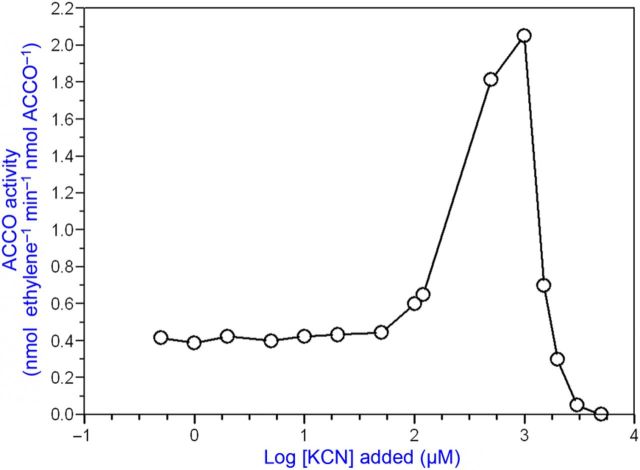
Cyanide activates ACCO. Cyanide activation begins at 100 µM, is maximal at ca. 0.5 mM and becomes inhibitory beyond 1 mM. One nmole of ACCO (1 µM in standard assay) was treated with KCN at the indicated final concentrations employing the standard assay for the fusion protein.

**Figure 12.**
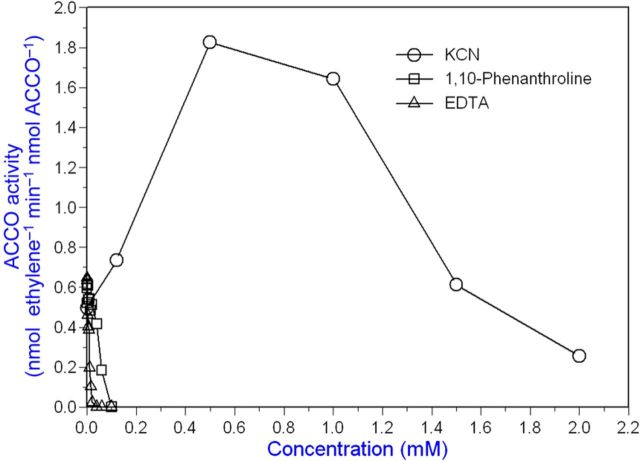
Cyanide activates ACCO beginning at ca. 100 µM and becomes inhibitory beyond 1 mM, whereas iron chelators inhibit activity at much lower concentrations. The ACCO fusion protein (1 µM in assay) was assayed in the standard assay with KCN, 1,10-phenanthroline or EDTA at the concentrations indicated. The iron chelators are strongly inhibitory at 0.1 mM, whereas cyanide up to 1 mM activates ACCO.

We conducted studies using alternative substrates and cofactors for ACCO, which catalyses the oxidation of amino acids other than ACC. α-Aminoisobutyric acid as a substrate produces acetone, ammonia and CO_2_ (but not cyanide) employing the same cofactors as ACCO with ACC as substrate (Charng *et al.* 1997). Since ACCO catalyses a reaction with AIB as substrate and cyanide is not produced, we determined whether cyanide has any effect on the reaction with ACC as substrate. When ACCO was incubated with cofactors, AIB and cyanide, and subsequently assayed with ACC as substrate, enzyme activity was retained/enhanced over the 2-h course of incubation, while enzyme activity decreased without AIB/cyanide (Fig. 10). Cyanide or AIB alone afforded partial protection (Ververidis and Dilley 1995). During the 2-h incubation with AIB/cyanide (while AIB was presumably oxidized to products), ACCO was not inactivated. ACC oxidase is activated by cyanide concentrations between 0.1 and 1 mM (Fig. 11). Moreover, these cyanide concentrations correspond to those generated during the 20-min enzyme assay at 20 °C based on the amount of ethylene produced and calculated from the data in Fig. 3. Beyond 1 mM, cyanide becomes inhibitory. Iron chelators such as EDTA and 1,10-phenanthroline at concentrations well below 100 µM strongly inhibit ACCO activity as a consequence of removing iron from the reaction centre (Fig. 12).

Rocklin *et al.* (2004) showed that ACCO is capable of doing a single turnover without Asc but only in the presence of bicarbonate and then becomes inactivated. They also introduced the notion of two Asc binding sites. One has a high affinity for Asc (Asc *K*_a_ = 0.2 mM) associated with initial activation of ACCO as an effector molecule, during which time Asc is not oxidized. Moreover, the effector role of Asc could be mimicked with the non-redox active Asc analogue saccharic acid 1,4-lactone. To gain further insights into a non-redox role of Asc in the ACCO reaction, we tested numerous structural analogues of Asc. We confirmed the results of Brunhuber *et al*. (2000) and Rocklin *et al*. (2004) showing that saccharic acid 1,4-lactone was a competitive inhibitor of ACCO with respect to Asc **[see Supporting Information]**. We found that 2,4-pteridinediol (lumazine) competitively activated ACCO with respect to Asc **[see Supporting Information]**. Plotting the Asc apparent *K*_a_ value vs. 2,4-pteridinediol concentration from the double reciprocal plot yielded a *K*_a_ = 2.2 mM similar to the Asc *K*_a_ of 2.58 mM.

ACC oxidase also produces ethylene with 2,4-pteridinediol without Asc but activity is only ∼14 % of that with Asc at equal concentration. 2,4,5-Triamino-6-hydroxypyrimidine competitively inhibited ACCO activity with respect to Asc **[see Supporting Information]**, while 4,5,6-triaminopyrimidine uncompetitively activated ACCO with respect to Asc (data not shown). l-Gulonic 1,4-lactone also uncompetitively activated ACCO (data not shown).

### Protein–protein interactions and phosphorylation studies with ACCO

Recombinant apple ACCO1 with a His-Tag bound to a Ni^2+^ column was employed as a protein affinity column to determine whether any proteins from apple fruit can bind to ACCO through protein–protein interactions (Dilley *et al.* 1995). Products of an *in vitro* phosphorylation assay conducted with a 100 000 *g* membrane fraction collected from pre- and postclimacteric apple fruits in the presence or absence of recombinant ACCO were passed through the His-Tag ACCO affinity column. The column was then eluted with imidazole. This would remove the ACCO along with proteins bound to ACCO from the phosphorylation assay. The eluate was subjected to SDS–PAGE analysis. A similar assay was done with ACCO only. Numerous proteins of unknown nature from both pre- and postclimacteric apple fruits became differentially phosphorylated (Fig. 15A and B). Moreover, ACCO became phosphorylated when assayed separately (Fig. 15A). Protein phosphorylation was more evident in postclimacteric as compared with preclimacteric fruit. A protein of ∼35 kDa became more heavily phosphorylated in postclimacteric as compared with preclimacteric fruit in the presence of ACCO. This 35-kDa protein is not a breakdown product of ACCO because it is absent in lane 3 (Fig. 15A). A strongly phosphorylated protein of ∼70 kDa was found when ACCO was assayed separately. This protein was not detectable by Coomassie Blue staining. There was no radiolabel at the mass of ACCO for pre- and postclimacteric fruit (Fig. 15A, lanes 2 and 5). The pattern of ^32^P labelling differences between pre- and postclimacteric fruit suggests maturity-dependent changes in kinase and phosphatase activities.

## Discussion

### ACC binding sites

ACC oxidase amino acids Cys28, Thr157, Lys158, Arg175, Gln188, Lys199, Arg244, Ser246, Lys230, Lys292, Glu294, Glu297, Arg299, Phe300 and Glu301 were found to be important residues affecting enzyme activity. Mutation of these amino acids differentially affects ACC *K*_m_, Asc *K*_a_ and bicarbonate *K*_a_ values. Arg175, Arg244 and Ser246 were determined to be important residues affecting ACC binding. The ACC *K*_m_ values of ACCO mutants Arg175Glu, Arg244Lys and Ser246Ala were 379, 175 and 277 µM, respectively, while activity was reduced 14-, 3- and 2-fold, respectively (Table 1). Arg175 mutants with side chains lacking a positive charge or H-bonding function had several-fold higher ACC *K*_m_ values and 15- to 42-fold lower activities compared with native ACCO. Lys158 (7.9 Å from the iron) mutants with a neutral or negative charge also exhibited elevated ACC *K*_m_ values, particularly as double mutants with Arg175 (Table 1). Bassan *et al.* (2006) proposed that Lys158 interacts with bicarbonate in activating the ACCO reaction. The single and double mutants of Lys158 have greatly reduced activities ranging from 14- to 1000-fold. We had proposed earlier that the ACC carboxyl group may interact with the Arg244 guanidinium group and the hydroxyl group of Ser246 (Dilley *et al.* 2003). However, the model proposed by Seo *et al.* (2004) and Yoo *et al.* (2006) has Arg244 and Ser246 interacting with Asc. The model proposed by Brisson *et al.* (2012) indicates Arg244, Ser246, Lys158 and Arg299 (apple ACCO numbering) interacting with bicarbonate H-bonded to the ACC carboxylate oxygen. However, with the exception of the Arg299Glu mutant (which has no measurable activity), the other Arg299 mutants have typical ACC *K*_m_ values. Bicarbonate interacting with Arg244 and Ser246 has been proposed to assist orientation of ACC at the ACCO/Fe(II)/ACC reaction site (Rocklin *et al.* 2004). The importance of Thr157 may be to stabilize β-strand 4 (Fig. 1) **[see Supporting Information]** containing Lys158 essential for the reaction since the Thr157Ala mutant ACC *K*_m_ was similar to that of native ACCO, whereas activity was reduced 17-fold (Table 1) and *V*_max_ was reduced 14-fold (Fig. 2). The triple-mutant Arg244Lys/Ser246Ala/Thr157Ala was virtually inactive (Table 1 and Fig. 2).

### Bicarbonate binding sites

Arginine and lysine (bearing a positive charge) are good candidates for interacting with bicarbonate. We found that bicarbonate rather than CO_2_ (gas) is the species involved in ACCO activation (Dilley *et al.* 2003). The eight conserved Lys residues of ACCO were mutated to other residues (Kadyrzhanova *et al.* 1999). Among these mutants, Lys158Leu, Lys199Glu, Lys230Glu and Lys292Glu were the least active, confirming the results of Charng *et al.* (1997). Mutants Arg175Glu, -Gln, -His, -Ala and -Gly all lack a positive charge needed to interact with bicarbonate and have greatly elevated bicarbonate *K*_a_ values (Tables 1 and 2). The Arg175Lys mutant (Lys is able to interact with bicarbonate) bicarbonate *K*_a_ = 6.3 mM is only slightly elevated over that of the native ACCO while its activity is reduced 5-fold, confirming results reported earlier (Dilley *et al.* 2001, 2003). The bicarbonate *K*_a_ of the Arg175Glu mutant was 10- to 16-fold higher than that of the native ACCO (Table 1 and Fig. 4). Bicarbonate activation of the Arg175Glu mutant was non-saturable (linear vs. hyperbolic), indicating a lack of a specific binding site (Fig. 4). The Arg175Gln mutant (lacking a positive charge) activity was also linear with bicarbonate concentration (Fig. 4). This is consistent with bicarbonate binding by H-bonding/electrostatic interactions with the Arg175 guanidinium group serving as a general base to abstract H-atoms from the Lys158 amino group as proposed by Bassan *et al.* (2006) forming the iron peroxo (ACCO/Fe(III)–OOH) complex as proposed by Rocklin *et al.* (2004). Several amino acid side chains in ACCO apparently provide an extensive network of H-bonds to support acid/base stabilization of the transition state complex. The bicarbonate *K*_a_ of native ACCO is 3.1 mM, while that for the Arg175Glu mutant is 51 mM (Table 1). Alternatively, Arg175 may serve an important structural role in bicarbonate activation. The studies of Brisson *et al.* (2012) show the backbone carbonyl O of Arg175 H-bonding to the Asc C_2_-OH group but the guanidinium group was not implicated as a bicarbonate binding site. Their studies show Arg244, Ser246, Lys158, Arg300 (tomato) and Tyr162 H-bonding with bicarbonate and bicarbonate H-bonding with the ACC carboxyl group. They also show Arg300 H-bonding with the C_1_ carbonyl O of Asc. The Yoo *et al.* (2006) model shows the Arg299 guanidinium group H-bonding with bicarbonate. Additional evidence for the role of Arg175 was gained from experiments with 1,2-cyclohexanedione (1,2-CHD) that (reportedly) specifically and reversibly modifies arginyl residues (Patthy and Smith 1975*a*, *b*). Following the Patthy and Smith experimental protocol, we found that 1,2-CHD inhibited native ACCO activity competitively with respect to bicarbonate raising the bicarbonate *K*_a_ 4-fold but did not inhibit the activity of the Arg175Glu and Arg175Gln mutants (data not shown). The Arg175Lys mutant activity was inhibited by 1,2-CHD by about the same degree as that of the native ACCO, suggesting that the reagent may also have some affinity for the Lys epsilon amino group. The Arg175Lys mutant has significant activity and near-normal bicarbonate *K*_a_ (Table 1). Arg175Glu mutant activity was 14-fold lower than that of native ACCO (Table 1). The 7-fold increase in the ACC. The *K*_m_ of the Arg175Glu mutant may be related to the proposed role of Arg175 as a bicarbonate binding site assisting ACC binding to ACCO/Fe(II). The data are consistent with bicarbonate binding by H-bonding/electrostatic interactions with an Arg guanidinium group as for Cu,Zn-SOD (Sankarapandi and Zweir 1999). The Thr157Ala mutant has a very high affinity for bicarbonate (*K*_a_ = 0.57 mM). This is nearly 9-fold greater than that of the native enzyme (*K*_a_ = 4.9 mM) and greater than that of all the other mutants studied (Table 1) with the exception of the Phe187Tyr mutant (Table 2). The activity of the Thr157Ala mutant is only ca. 6 % that of the native enzyme. Lys158 is also an important binding site for bicarbonate based on greatly elevated bicarbonate *K*_a_ values for Lys158Gln, Lys158Glu and Lys158Leu mutants, particularly as double mutants with Arg175 (Table 1). Such large apparent bicarbonate *K*_a_ values indicate a lack of a binding site (linear vs. hyberbolic kinetics). The ACCO activity of these mutants was reduced 32- to 1000-fold. Removing the Arg175 and Lys158 bicarbonate binding sites virtually eliminates ACCO activity. Arg299 of apple ACCO is equivalent to Arg300 in tomato ACCO and Brisson *et al.* (2012) proposed Arg300 as a binding site for bicarbonate in addition to Arg244, Ser246 and Lys158. Our results largely support the modelling studies of Brisson *et al*. (2012), implicating these residues as bicarbonate binding sites. However, we find that ACCO mutants Arg299Leu and Arg299His have ACC *K*_m_ and bicarbonate *K*_a_ values similar to those of native ACCO, whereas activity is reduced 90- to 400-fold (Table 1). The Arg299Lys mutant ACC *K*_m_, Asc *K*_a_ and bicarbonate *K*_a_ values were similar to those of the native enzyme and the activity was only reduced ca. 2-fold (Table 1). The nearly equal kinetic parameters of the Arg299Lys mutant (bearing an ε-amino group) and native ACCO (bearing a guanidinium group) indicate the importance of a positively charged residue for interacting with bicarbonate. The Arg299Glu mutant (bearing a carboxylate group) cannot interact with bicarbonate and has no measurable activity (Table 1). Bassan *et al.* (2006) concluded (from density functional theory (DFT) calculations) that the most likely role of bicarbonate in the catalytic reaction of ACCO is to facilitate a proton transfer from the ε-amino group of Lys158 to the leaving OH group during the activation of ACCO/Fe(III)═O-OH to ACCO/Fe(IV)=O. Lys158 is ∼7.9 Å from the iron atom (Zhang *et al.* 2004). The close proximity of positively charged Lys296 and Arg299 from α-helix 11 at the reaction centre would likely lower the p*K*_a_ of Lys158 sufficiently for this to occur (Schmidt and Westheimer 1971; Highbarger *et al*. 1996). Yoo *et al.* (2006) found that both Lys296 and Arg299 (apple) are essential for ACCO activity, while our more limited studies show that the Lys296Glu mutant has near-normal activity (Table 2). Phe300 is also important for ACCO activity. The Phe300Gln mutant had very low activity and was not activated by bicarbonate at concentrations <2 mM (Fig. 8) and higher concentrations exhibited non-saturation. It exhibited a nearly 3-fold higher ACC *K*_m_ and a 4.4-fold lower Asc *K*_a_ (Table 2). Phenylalanine would not be a binding site for bicarbonate but is required for ACCO activation. The Phe300Tyr mutant was 36-fold more active than the Phe300Gln mutant and exhibited normal values for ACC *K*_m_, and bicarbonate and Asc *K*_a_. Arg175, Arg299 and Lys158 play important roles as binding sites for bicarbonate in the ACCO reaction. Lys158 and Phe300 may also be binding sites for Asc as described below.

### Ascorbic acid binding sites

Ascorbic acid binds in proteins by salt bridges, H-bonding and hydrophobic interactions (Burmeister *et al.* 2000; Li *et al.* 2001). Seo *et al.* (2004) provided evidence that Arg244 and Ser246 (the Arg–X–Ser motif) interact with Asc through H-bonding. The studies of Brisson *et al.* (2012) indicate a role for these Arg and Ser residues in H-bonding interactions with bicarbonate. Collectively, these studies shed doubt on ACC interacting directly with Arg244 and Ser246, although mutants of these residues exhibit an elevated ACC *K*_m_ as we show in Fig. 2 and Table 1. Ascorbate can also form a Schiff base with the Lys ε-amino group (McLaughlin *et al.* 1980; Larisch *et al.* 1996; Lee *et al.* 1998). Arginine and glutamine can also form Schiff bases with Asc analogues (Morris *et al.* 1996). In the Morris *et al.* (1996) study, a Schiff base was formed between the active-site Lys146 ε-amino group and the carbonyl carbon of the ketose substrate of rabbit aldolase. And, the Lys146Arg mutant of rabbit aldolase was active but at a slower rate. This suggests that perhaps the Asc C_1_ carbonyl carbon may also form a Schiff base with an active-site lysyl residue in ACCO. However, it is not likely that Asc forms a Schiff base in its redox active role. It may, however, do so in the non-redox role of Asc as an effector molecule (Rocklin *et al.* 2004). Direct evidence needed to demonstrate a Schiff base complex between Asc and a lysyl residue would require using sodium borohydride to stabilize the complex and performing subsequent peptide and amino acid analyses to prove the identity of the lysyl residue. Lys158 and Lys292 appear to be most critical for Asc activation and these may be the lysyl residues modified by the lysine-specific reagents (Fig. 5) **[see Supporting Information]** to explain PLP inhibition. Perhaps Asc may form a Schiff base with a lysyl residue in ACCO in its role as an effector molecule. This warrants further investigation. The ε-amino group of Lys292 is implicated as a binding site for Asc based on inability of the Lys292Glu mutant to be activated by Asc (Fig. 7). The extremely low residual activity of the Lys292Glu mutant may represent single turnovers of ACCO, after which ACCO becomes inactivated without Asc serving its normal role as a reductant during reaction turnover. Rocklin *et al.* (2004) conducted experiments showing that ACCO is capable of doing a single turnover without Asc (but only in the presence of bicarbonate) and subsequently is inactivated. They also introduced the notion of two Asc binding sites. One site has high affinity for Asc (*K*_a_ = 0.2 mM) and is associated with initial activation as an effector molecule, during which time Asc is not oxidized, while ACCO/Fe(II)–O–O/ACC is oxidized to ACCO/Fe(III)═O–OH/ACC. Mirica and Klinman (2008) have questioned the need of an effector role for Asc based on their investigations. In any event, the effector role of Asc can be mimicked by the non-redox active Asc analogue saccharic acid 1,4-lactone (Rocklin *et al.* 2004). We find that saccharic acid 1,4-lactone is a competitive inhibitor with respect to Asc **[see Supporting information]**, confirming the results of Brunhuber *et al.* (2000) and Rocklin *et al.* (2004). 2,4-Pteridinediol (lumazine) competitively activates ACCO with respect to Asc **[see Supporting Information]**. Plotting Asc *K*_a_ apparent vs. 2,4-pteridinediol concentration from a double reciprocal plot of data from Fig. S5 **[see Supporting Information]** yields a 2,4-pteridinediol *K*_a_ = 2.2 mM, which is similar to the Asc *K*_a_ of 2.58 mM. ACCO also produces ethylene with 2,4-pteridinediol in the absence of Asc but activity is only ca. 14 % compared with the same levels of Asc (data not shown). 2,4,5-Triamino-6-hydroxypyrimidine competitively inhibited ACCO activity with respect to Asc **[see Supporting Information]**, while 4,5,6-triaminopyrimidine uncompetitively activated ACCO with respect to Asc (data not shown). l-Gulonic 1,4-lactone also uncompetitively activated ACCO (data not shown). These results provide some evidence that there may be a binding site for Asc in a non-redox active role. This non-redox role may be as a base catalyst. Although quite speculative, perhaps this Asc may form a Schiff base with one of the two essential lysyl residues in ACCO based on our results using lysine-specific reagents and competitive inhibition of ACCO by PLP. Moreover, the study by Brunhuber *et al.* (2000) (their fig. 5) indicates uncompetitive activation of ACCO by Asc. Shikita *et al.* (1999) have described uncompetitive activation of myrosinase by Asc in a non-redox role wherein it serves as a catalytic base or nucleophile (Ettlinger *et al.* 1961). Burmeister *et al.* (2000) reported that Asc is a cofactor for myrosinase and serves as a catalytic base to activate a water molecule without Asc becoming oxidized. Binding sites for Asc in myrosinase were determined by X-ray crystallography to include an Arg residue guanidinium group interacting with Asc C_1_-O and C_2_-OH. Brisson *et al.* (2012) proposed that the tomato ACCO Arg300 (apple Arg299) guanidinium group interacts with the Asc C_1_-O, and the Arg175 main chain carbonyl oxygen interacts with the Asc C_2_ hydroxyl group. Thus, the non-redox ‘effector role’ of Asc as proposed by Rocklin *et al.* (2004) may promote protein structure changes or serve as a base catalyst in the ACCO reaction.

We find that Arg299 and Arg175 are important for activity of ACCO but Arg299Lys, Arg299His and Arg299Leu mutants have *K*_m_ values for ACC and *K*_a_ values for bicarbonate similar to those of the native ACCO, while the Asc *K*_a_ values for the Arg299Leu and Arg299His mutants are greatly reduced (Table 1). The results suggest that the positive charge of the Arg299 guanidinium group may be an important binding site for Asc. This would be consistent with the modelling studies of Brisson *et al*. (2012) showing that this Arg residue H-bonds with bicarbonate and also to Asc. The Arg299Glu mutant with a negative charge would not be a binding site for either Asc or bicarbonate and has no measurable activity (Table 1).

**Scheme 1.**
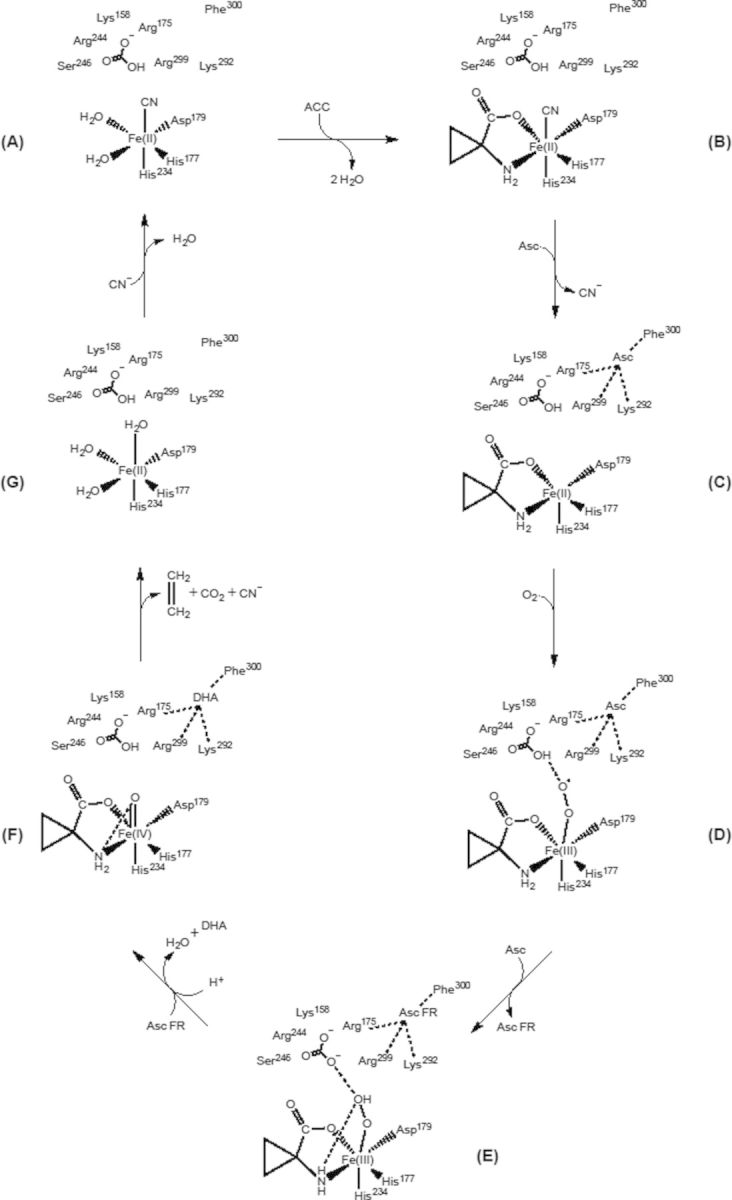
ACCO reaction mechanism modelled after Mirica and Klinman (2008). The reaction cycle is consistent with our steady-state kinetic data for mutants of Arg175, Lys158, Arg299, Lys292 and Phe300 as bicarbonate and Asc binding sites. We propose that Asc reduces ACCO/Fe(III)═O–O to ACCO/Fe(III)=O–OH producing the ascorbate free radical. Ascorbate (or the ascorbate free radical) and bicarbonate in the outer sphere interact with ACCO/Fe(III)=O-OH at the rate-determining step E to F forming ACCO/Fe(IV)=O.

The second binding site for Asc is required for multiple reaction turnovers. The Lys292Glu mutant is not activated by Asc, and PLP does not inhibit its low residual activity (Fig. 7). Pyridoxal-5′-phosphate inhibits the native ACCO ostensibly by forming a Schiff base with an essential Lys-ε-amino group, and our data indicate that this is Lys292. Pyridoxal-5′-phosphate competes with ascorbate for the Asc binding site (Fig. 6) **[see Supporting Information]**. The data from Fig. 3 yield a PLP *K*_i_ = 0.2 mM. This indicates that Lys292 may be an important binding site for Asc. Other lysine mutants were all competitively inhibited by PLP including Lys158Arg, Lys172Glu, Lys199Glu and Lys230Glu (data not shown), and this is consistent with Lys292 being the Asc binding site. Lys199 is at the C-terminus of β-strand 7 (Fig. 1) **[see Supporting Information]** placing it near the reaction site (14.1 Å from the iron atom), and when mutated to glutamate (Lys199Glu) ACCO activity is reduced ∼100-fold (Table 2). The Lys199Glu mutant has a normal bicarbonate *K*_a_ but a greatly reduced *K*_a_ for Asc (*K*_a_ = 0.54 mM). The *p*K_a_ of the Lys292 ε-amino group, when positioned among the positively charged amino acids (Lys296, Arg299 and Lys304) from the C-terminal α-helix-11 **[see Supporting Information]** along with Lys158 and Lys199 in close proximity to the reaction centre, would be expected to be lowered sufficiently (Schmidt and Westheimer 1971; Highbarger *et al.* 1996) to promote interaction with PLP or Asc. The studies of Brisson *et al.* (2012) show Arg300 (apple Arg299) to be H-bonded to both bicarbonate and the Asc C_1_ carbonyl oxygen. ACCO activity is markedly increased when Pro298 in α-helix 11 is mutated to Ala, which would favour a more linear α-helix **[see Supporting Information]** that may position Lys292 and Arg299 more favourably for reactivity. The Pro298Ala mutant has the same ACC *K*_m_ as the native enzyme (ca. 60 µM) and the same bicarbonate *K*_a_ of 3.1 mM, while the Asc *K*_a_ is only reduced from 2.3 to 1.7 mM (Table 2). Interestingly, the Pro298Ala mutant exhibited more activity than the native enzyme when carbon dioxide was scrubbed from the assay medium. We reason that the high activity of the Pro298Ala mutant may be due to stabilizing the H-bonding network and electrostatic interactions involving ACC, Asc, bicarbonate and amino acid side chains of Lys158, Arg175, Arg299, Lys292, Arg244 and Ser246. This would be consistent with the high value of the ^18^O KIE of 1.0215 found for ACCO (Mirica and Klinman 2008). This has been attributed to outer sphere reorganization of the ‘players’ leading to lowering of the reaction activation barrier (Roth *et al.* 2004). Other amino acids in α-helix 11 and the loop leading to the helix are important for ACCO activity. These include Glu294, Glu297, Glu301 and Phe300 (Table 2). The kinetic parameters for the Phe300Gln mutant are noteworthy because of the low Asc *K*_a_ of 0.52 mM and the 32 mM *K*_a_ for bicarbonate (non-saturable). The activity of the Phe300Gln mutant is reduced >200-fold. The Phe300Gln mutant shows no activation by bicarbonate at concentrations <2 mM. The phenylalanine side chain does not bind bicarbonate, although the amide group of the Phe300Gln mutant may H-bond with bicarbonate. The Phe300Tyr mutant has a more typical bicarbonate *K*_a_ of 6.5 mM and is ca. 7 times more active than the Phe300Gln mutant (Table 2). Phe300 may interact with Asc and help binding of bicarbonate as suggested by the Brisson *et al.* (2012) studies. The phenyl ring of Phe300 or Phe300Tyr mutants may stabilize Asc through hydrophobic interactions with the planar 1,4-lactone ring of Asc. This type of interaction has been shown for Asc in hyaluronate lyase (Li *et al.* 2001). Other residues (Arg, Asn and Tyr) in hyaluronate lyase were shown to interact with Asc. A binding site for Asc in ascorbate peroxidase also involves an Arg residue (Arg172) where the guanidinium group interacts with the Asc C_2_-OH, C_3_-OH and C_6_-OH groups (Sharp *et al.* 2003). The Asc activation profile of the Phe300Gln mutant is similar to the bicarbonate activation profile of the Lys158Leu mutant (Figs 8 and 9). This suggests that Asc and bicarbonate may participate in the same rate-determining step (Scheme 1D to E) with some binding sites in common. Studies of oxaloacetic acid (OAA) inhibition of ACCO support this concept. Oxaloacetic acid is a competitive inhibitor of ACCO with respect to Asc (*K*_i_ = 0.24 mM) and a non-competitive inhibitor with respect to ACC and bicarbonate (Iturriagagoitia-Bueno *et al.* 1996). We have confirmed that OAA inhibition is competitive with respect to Asc (but not with ACC) and determined the OAA *K*_i_ to be 0.93 mM (data not shown). Malonic acid is also a competitive inhibitor with respect to Asc acid with *K*_i_ = 0.07 mM (data not shown). Both OAA and malonic acid would neutralize the positive charge of a lysyl or arginyl residue and explain the inhibition. The Lys158Leu mutant is not inhibited by OAA at 2 mM OAA (data not shown), supporting the role of the Lys158 ε-amino group as a binding site for Asc.

We propose that Asc (or ascorbate free radical, AFR) bound to Lys292, Lys158 and Phe300 activates the ACCO/Fe(III)–OOH/ACC/HCO_3_ complex to ACCO/Fe(IV)=O)/ACC in cooperation with bicarbonate interacting with Lys158, Lys175 and Arg299 (Scheme 1D and E). Reaction site models (Yoo *et al.* 2006; Brisson *et al.* 2012) that place these residues in close proximity to the iron centre together with the results of site-directed mutagenesis studies (Seo *et al.* 2004; Yoo *et al.* 2006) and the results of studies reported herein provide chemical insights into how these amino acids bind and activate ACCO to produce ethylene. Yoo *et al.* (2006) found that Lys296 of apple ACCO is essential for activity, but our more limited studies show that Lys296Ala is slightly more active than the native ACCO.

### Cyanide activates and protects ACCO from inactivation

ACC oxidase catalyses oxidation of amino acids other than ACC. It is a d-amino acid oxidase and converts d-valine to *iso*-butanal (Gibson *et al.* 1998). α-Aminoisobutyric acid is also a substrate producing acetone, ammonia and CO_2_ (but not cyanide) employing the same cofactors as ACCO with ACC as substrate (Charng *et al.* 1997). It is also a competitive inhibitor (*K*_i_ = 0.3 mM) with respect to ACC (McGarvey and Christoffersen 1992). When ACCO was incubated with cofactors AIB and cyanide and subsequently assayed with ACC as substrate, enzyme activity was retained/enhanced over the 2-h course of incubation while enzyme activity decreased without AIB/cyanide (Fig. 10). During the 2-h incubation with AIB/cyanide (while AIB was presumably oxidized to products), ACCO was not inactivated. This suggests that the presence of cyanide with substrate prevents inactivation and may improve catalytic competency of the Fe-centred reaction (Ververidis and Dilley 1995). Bicarbonate also affords protection of ACCO from inactivation (Barlow *et al.* 1997; Zhou *et al.* 2002). When ACCO was pretreated with bicarbonate and subsequently assayed without supplemental bicarbonate, activity was comparable to that observed with supplemental bicarbonate (Fig. 3). The presence of cyanide as a ligand to ACCO/Fe(II) may assist ACC binding, as proposed for bicarbonate. ACCO/Fe(II) and ACCO/Fe(II)/HCO_3_^−^ are 6-coordinate and bicarbonate bound to Arg175 interacting with ACCO/Fe(II) would account for the NIR CD titration results of Zhou *et al.* (2002). We propose that cyanide becomes an iron ligand during reaction turnover as shown in Scheme 1A. There is precedence for cyanide to become an iron ligand of protocatechuate 3,4-dioxygenase (PCD), which is also a non-haem (Fe(III)) enzyme (Whittaker and Lipscomb 1984; Orville *et al.* 1997). However, ACCO is an Fe(II) enzyme but cyanide also binds to Fe(II). Cyanide readily displaces water as an iron ligand. In PCD, cyanide (as an oxygen surrogate) binds *trans* to a histidine ligand as an equatorial iron ligand. Substrate binding precludes cyanide binding. We propose that cyanide becomes an ACCO/Fe(II) ligand *trans* to His234 during completion of reaction turnover and thus precedes binding of the ACC substrate and oxygen (Scheme 1A). Under standard assay conditions, ACCO is activated by cyanide concentrations between 0.1 and 1 mM (Fig. 11). Moreover, these cyanide concentrations correspond to those generated during the 20-min enzyme assay at 20 °C (data not shown). *V*_max_ without supplemental cyanide is achieved after ∼20 min as a consequence of cyanide accumulation during reaction turnover to ∼150 µM in the assay as calculated from the data in Fig. 3. Beyond 1 mM, cyanide becomes inhibitory. Iron chelators such as EDTA and 1,10-phenanthroline at concentrations well below 100 µM strongly inhibit ACCO activity as a consequence of removing iron from the reaction centre (Fig. 12). Oxygen binds only to ACCO/Fe(II). Oxygen would readily displace cyanide as an Fe(II) ligand since the *K*_m_ for oxygen is ∼28–67 µM (Thrower *et al.* 2001; Mirica and Klinman 2008). Alternatively, we propose that Asc may displace cyanide as an iron ligand as shown in Scheme 1B and C. l-Ascorbic acid (as the ascorbate free radical AFR) may activate ACCO by interacting with the ternary complex ACCO/Fe(III)/ACC/OOH/HCO_3_ since high Asc levels become inhibitory by interacting with the enzyme/substrate complex (Segal 1993).

Cyanide derived as a product of the ACCO reaction may be sufficient to cause similar effects *in vivo* before the mitochondrial enzyme β-cyanoalanine synthase can detoxify it (Peiser *et al.* 1984). Other roles for ACCO-derived cyanide in plant metabolism have been found or implicated. Grossman and Kwiatkowski (1995) demonstrated that cyanide derived from ACC from herbicide-stimulated ACC synthase activity is a causative factor in the herbicidal effects observed in shoots of barnyard grass (*Echinochloa crus-galli*). Cyanide levels of 40 µM were found in shoot tissues of quinclorac-treated plants, and cyanide is a potent inhibitor of cytochrome oxidase. Nearly four decades ago, Solomos and Laties (1975) observed that cyanide triggered ethylene production and induced the cyanide-insensitive respiratory pathway in potatoes. More than two decades ago, Pirrung and Brauman (1987) presented a hypothesis connecting ethylene and cyanide effects in plant physiology. Bisson and Groth (2010) found that cyanide (an ethylene agonist) increases the affinity of the ethylene receptors for EIN2. Cyanide, as a product of the ACCO reaction, may be involved in diverse metabolic pathways.

### ACCO reaction mechanism

Numerous models of the ACCO reaction mechanism have been proposed (Rocklin *et al.* 1999, 2004; Zhou *et al.* 2002; Solomon *et al.* 2003; Hausinger 2004; Mirica and Klinman 2008). The model of Mirica and Klinman (2008) is most consistent with the available data as shown in Scheme 1. Their model considers ACC to bind to ACCO/Fe(II) like α-ketoglutarate (αKG) binds to other αKG-dependent non-haem iron enzymes. The study of Ye *et al.* (2012) provides an excellent analysis of the reaction mechanism of αKG-dependent enzymes. Models of the ACCO reaction centre presented by Seo *et al.* (2004), Yoo *et al.* (2006) and Brisson *et al.* (2012) suggest specific amino acids as binding sites for ACC and bicarbonate and Asc cofactors that activate ACCO. We have modified the model of Mirica and Klinman (2008) to indicate the role of specific amino acid residues involved in binding of bicarbonate and Asc based on our site-directed mutagenesis studies and we have shown how some of these amino acids interact with the reaction centre from modelling studies. We also propose that cyanide becomes a ligand of ACCO/Fe(II) after reaction turnover (Scheme 1A). This model is in agreement with that of Mirica and Klinman (2008) and is supported by oxygen activation studies of non-haem iron complexes (Lee *et al.* 2010; Ye *et al.* 2012). ACC binding to ACCO precedes oxygen binding (Thrower *et al.* 2001). Spectroscopic studies (Zhou *et al.* 2002) reveal that when the ACCO/Fe(II)/ACC/HCO_3_ complex is titrated with Asc it converts from a 6-coordinate (octahedral) to a 5-coordinate (square pyramidal) geometry, thereby providing the site for oxygen to bind. We propose that Asc may serve as an effector molecule displacing the cyanide ligand (Scheme 1B to C). This would account for the formation of the 5-coordinate geometry needed for oxygen to bind. When oxygen binds to this complex, it becomes 6-coordinate. Which leads to oxygen activation. Tierney *et al.* (2005), employing NO as a surrogate for oxygen, provided evidence that binding of ACC dictates the orientation of bound oxygen to be *trans* to one of the histidine ligands. This is consistent with the modelling studies conducted by Brisson *et al.* (2012) showing oxygen bound *trans* to His234. Ascorbate (by an outer sphere mechanism) reduces the ACCO/Fe(III)O=O/ACC/HCO_3_ complex to the ACCO/Fe(III)=OOH/ACC/HCO_3_ complex with formation of the AFR (Scheme 1D to E). Ascorbate provides the electron to form the intermediate ACCO/Fe(IV)
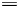
O/ACC/HCO_3_ (Scheme 1E to F), which is the rate-limiting step (Mirica and Klinman 2008). Density functional theory analysis of the ACCO reaction revealed the same rate-limiting step (Bassan *et al.* 2006). The AFR is relatively unreactive (Bielski 1982). This may account for Scheme 1E to F to be the rate-limiting step in the reaction. Alternatively, two AFRs may disproportionately yield Asc and dehydroascorbate, which is the general fate of the AFR (Bielski 1982). This would allow Asc rather than the AFR to be the reductant (Scheme 1E to F). The net result would be the same stoichiometrically, namely one Asc yielding one dehydroascorbate leading to the formation of the iron-oxo complex (Scheme 1F). The iron–oxo intermediate (F) would abstract the H atoms from the ACC amino group and rapidly fragment into ethylene and cyanoformate as the iron–oxo oxygen atom is reduced to water. Cyanoformate is unstable and breaks down to cyanide and CO_2_ (Peiser *et al.* 1984). At the completion of reaction turnover, we propose that cyanide becomes an ACCO/Fe(II) ligand *trans* to the His234 ligand (or *trans* to His177) with two water molecules as ligands, as shown in Scheme 1A. Cyanide readily replaces water as an Fe(III) ligand as found for protocatechuate 3,4-dioxygenase (Whittaker and Lipscomb 1984; Orville *et al.* 1997). However, ACCO is an Fe(II) enzyme. Cyanide can bind to both Fe(II) and Fe(III) (Clay *et al*. 2002; Leavesley *et al.* 2008). Cyanide is a stronger ligand to Fe(III) than to Fe(II), but if bound to Fe(III) this would limit the ability of Asc or the AFR to reduce oxygen (Shearer *et al.* 2003). Dioxygen binding to the complex ACCO/Fe(II)/ACC/Asc (without bicarbonate) results in rapid inactivation of ACCO (Barlow *et al.* 1997; Zhou *et al.* 2002). Inactivation of ACCO is largely spared in the presence of bicarbonate and/or cyanide (Ververidis and Dilley 1995). This may promote a proton-coupled electron transfer (Shook and Borovick 2010) from the ACC amino group leading to the abstraction of a hydrogen atom. If the AFR is the reductant, it would provide only the electron since it is not protonated (Scheme 1E and F). Moreover, the ^18^O KIE decreases from 1.0215 ± 0.0005 at a saturating Asc concentration (20 mM) to 1.0157 ± 0.0004 at low Asc levels (2 mM). This observation together with the large 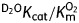 value suggests that the concentration of the AFR may be rate limiting. The role of bicarbonate would be to assist the formation of an intermediate complex (Scheme 1D to E) ACCO/Fe(III)═O═O═H involving Asc as the reductant leading to formation of the first water. The large ^18^O KIE value of 1.0215 ± 0.0005 determined for ACCO is consistent with an outer sphere electron transfer lowering the O═O bond order occurring at the rate-determining step (Mirica and Klinman 2008). The unstable iron–oxo intermediate (Scheme 1F) would break down to yield ethylene, CO_2_, HCN and H_2_O as Fe(IV) is reduced to Fe(II) with H atoms from the ACC amino group. Cyanide is a much stronger iron ligand than water particularly when positioned *trans* to a histidine imidazole nitrogen due to the strong *trans* effect of cyanide (Basolo and Pearson 1967) and so may become an iron ligand as shown in Scheme 1A. The scheme is generally consistent with the role of the amino acid residues shown clustered around the reaction site as bicarbonate and Asc binding sites as modelled by Brisson *et al.* (2012) and Yoo *et al.* (2006) and our steady-state kinetic analysis of ACCO mutants of many of the same residues (Kadyrzhanova *et al.* 1997, 1999; Dilley *et al.* 2001, 2003) and data presented herein.

### ACC oxidase fragmentation

**Figure 13.**
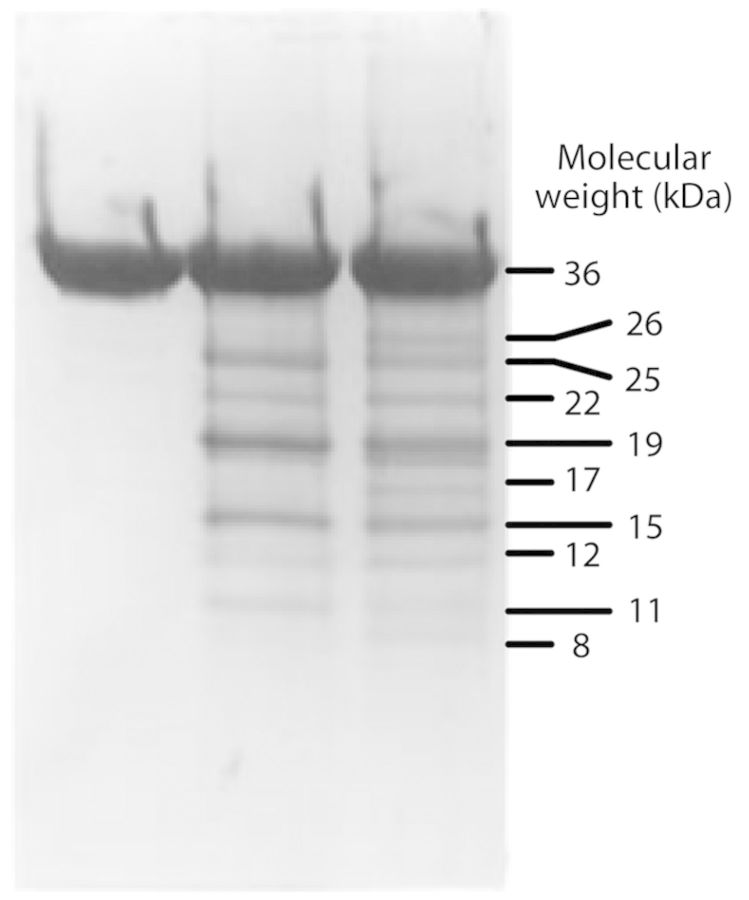
ACC oxidase becomes fragmented into polypeptides ranging in size from 8 to 26 kDa when assayed in the presence of oxygen, Fe(II) and Asc with or without ACC. Left lane, buffer only; middle lane, Fe(II) and Asc; right lane, Fe(II), Asc and ACC. The C-terminal 15-kDa fragment has the N-terminal sequence F187QDNKVS. The C-terminal 12-kDa fragment has the N-terminal sequence V215NLGD. All the other fragments have the same N-terminal sequence as intact ACCO. (Reprinted with permission from Zhang *et al.* (1997). Metal-catalysed oxidation and mutagenesis studies on the iron(II) binding site of 1-aminocyclopropane-1-carboxylate oxidase, *Biochemistry* **36**:15999–16007, Copyright 1997 American Chemical Society.)

ACC oxidase becomes rapidly inactivated during enzyme assays unless the ACCO/Fe(II) ratio is kept near unity with adequate bicarbonate present (Rocklin *et al.* 2004; Mirica and Klinman 2008) or unless the assay includes AIB and cyanide protection (Ververidis and Dilley 1995; present study). Fragmentation studies with tomato ACCO1 conducted with Fe(II)/ACCO ratios greater than one reveal two primary cleavage sites: one between Leu186 and Phe187, and the other between Val214 and Val215 yielding ca. 15- and 12-kDa polypeptides, respectively (Barlow *et al.* 1997; Zhang *et al.* 1997). These sites are not close enough to the catalytic iron site to allow a highly reactive oxygen species to directly cause cleavage (Zhang *et al.* 2004). Moreover, peptide bond cleavage occurs in the presence of Asc, ferrous ion and oxygen without ACC and bicarbonate. Hydrogen peroxide is known to promote peptide bond cleavage (Stadtman and Levine 2003), so an additional ferrous ion binding site may be present in ACCO that produces hydrogen peroxide in the presence of Asc and oxygen in a Fenton-type reaction. The presence of catalase protects against fragmentation (Smith *et al.* 1994). The polypeptides produced include, in addition to the 15- and 12-kDa C-terminal fragments, other polypeptides all with the same N-terminal amino acid sequence as the wild-type ACCO (Barlow *et al.* 1997; Zhang *et al.* 1997). These include polypeptides of 26, 25, 22, 19, 17, 11 and 8 kDa (Fig. 13). Polypeptides larger than 15 kDa suggest that proteolytic activity may be promoted by the initial fragmentation into 15- and 12-kDa polypeptides. Fragmentation requires oxygen and occurs with Asc without added ferrous ion and with ferrous ion without Asc. This suggests that some fragmentation occurs at the catalytic iron site and also at a second site. Since all the N-terminal fragments have the same N-terminal amino acid sequence as wild-type ACCO, further fragmentation may result from active oxygen species generated from Asc oxidation. Alternatively, ACCO has high homology with *Lycopersicon esculentum* cysteine protease LeCp (Table 3) and this may partially explain some of the fragmentation results. ACCO1 of apple has three conserved cysteine residues (Cys28, Cys133 and Cys165) and all are in the N-terminal fragments derived from cleavage at Leu186 and Val214. Among these, only Cys28 is essential for ACCO activity based on the very low activity of the Cys28Ala mutant (Table 2). The Cys28 sulfhydryl group must be present for enzyme activity. Cys28 is predicted to be close to the Leu186 and Val214 fragmentation sites according to the X-ray structure of ACCO from petunia (Zhang *et al.* 2004). ACC oxidase Cys28 is a putative cysteine protease active site in the sequence ACCO Asp–Ala–Cys28–Glu (Table 3), which may provide a metal binding site for cysteine protease activity similar to that of *L. esculentum* cysteine protease LeCp (Matarasso *et al.* 2005).

**Table 3.** ACC oxidase contains putative amino acid sequence motifs for post-translational modifications.

*N*-glycosylation site
Consensus: Asn–X–Ser/Thr
ACCO: Asn99-Ile–Ser
*O*-glycosylation site
Consensus: Ser–X–X–Pro
ACCO: Ser101–Glu–Val–Pro
Protein consensus sequence motifs in ACCO
Membrane protein sorting signal: Tyr–X–X–Hydrophobic (Hyd is Leu, Ile, Met or Phe)
ACCO: Tyr277–Pro–Lys–Phe
ACCO: Tyr287–Met–Lys–Leu
ACCO: Tyr291–Ala–Gly–Leu
All in the C-terminal polypeptide proteolytic fragment Val266–Met309
Phosphorylation site consensus sequences
Serine/threonine kinases
Cyclic-AMP-dependent kinase
Arg–X–X–Ser/Thr–(Hydrophobic)
ACCO: Arg–Ala–Ala–Thr20–Met
Ca-calmodulin-dependent protein kinase II
Arg–X–X–Ser/Thr–(Hydrophobic)
ACCO: Arg–Ala–Ala–Thr20–Met
Tyrosine protein kinase (Hydrophilic)_1–3_–X–Tyr–X–X– (Hydrophobic)
ACCO: Asp–Glu–Glu–Tyr110–Arg–Glu–Val (SH2 domain binding)
ACCO: Asp–Asp–Tyr287–Met–Lys–Leu (also membrane sorting signal)
ACCO: Asp–Trp–Glu–Ser–Tyr89–Phe–Phe–Leu
Protein–protein binding
SH2 domain BINDING (Src homology 2)
Consensus: Tyr^p^–X–X–Hydrophobic
ACCO: Tyr110–Arg–Glu–Val
ACCO: Tyr260–Pro–Ala–Pro
SH3 domain binding (Src homology 3)
Consensus: Pro–Pro–X–Pro
ACCO: Pro163–Pro–Cys–Pro
Coactivator motif (nuclear receptor transcriptional activation)
Consensus: Leu–X–X–Leu–Leu
ACCO: Leu–Leu–Asp–Leu–Leu
Transcriptional regulation motif
Leucine zipper
Consensus: Lys–X_6_–Leu–X_6_–Leu–X_6_–Leu–X_6_–Leu–X_6_–Leu
ACCO: Phe117–X6–Leu124–X_6_–Leu131–X_6_–Leu138–
Homology with cysteine proteases
LeCp: 197 SLVLYI E ACESGSI FE 212
++ + + ACE+ ++
ACCO: 20 TMEMIKDACENWGFFE 35

### Putative role of ACCO cysteine protease activity in ethylene signal transduction

**Figure 14.**
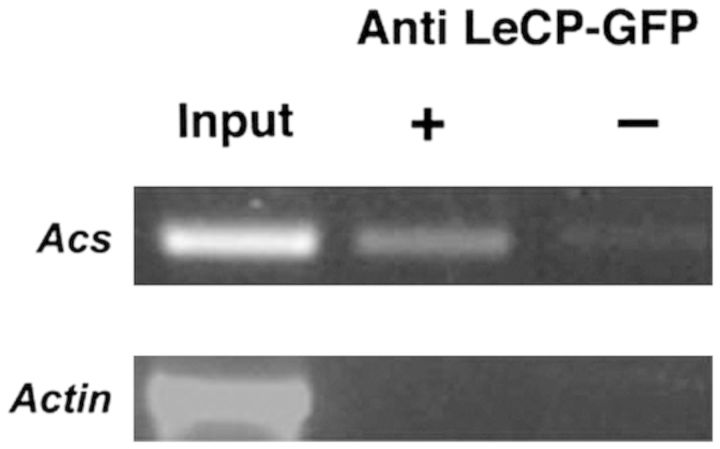
*In vivo* binding of *L. esculentum* cysteine protease (LeCp) protein fragment to the promoter sequence of the ACC synthase2 gene (*Acs2*) induces expression of *Acs2*. This was confirmed by PCR analysis using gene-specific primers of *Acs*2. (From: Matarasso *et al.* (2005). *Plant Cell* **17**:1205–1216, with permission from *Plant Cell*.)

Matarasso *et al.* (2005) described a novel plant cysteine protease (*L. esculentum* LeCp) that has a dual role as a protease and as a regulator of ACC synthase gene expression. First, LeCp acts as a protease in the cytoplasm. Then, upon elicitor signalling, a small ubiquitin-related protein binds to it, targeting it to the nucleus where it acts as a transcription factor inducing *LeAcs2* expression (Fig. 14). ACCO1 of petunia and apple have ∼30 % identity with *L. esculentum* LeCp. The active site cysteine in LeCp is Cys205 and this is equivalent to apple ACCO1 Cys28 (Table 3) **[see Supporting Information]**. The amino acid sequence Glu–Ala–Cys205–Glu in LeCp is similar to that of ACCO1 Asp–Ala–Cys28–Glu providing for a divalent metal binding site that is needed for protease activity. Other regions of LeCp and ACCO1 share about the same identities. It is conceivable that a portion of the ACCO protein may have ‘cysteine protease’ activity. This activity would be unrelated to the role of ACCO in catalysing the conversion of ACC to ethylene. Might a portion of the ACCO protein be modified in a manner similar to that found for LeCp in regulating some ethylene signal transduction pathway? This putative role for ACCO is highly speculative but is a researchable hypothesis. Cysteine proteases, among other proteases, also have transpeptidase (peptide ligase) activities providing for non-ribosomal assembly of polypeptides (Wang *et al.* 2008). They found a plant-specific papain-like protease (RD21) from *Arabidopsis thaliana* that accepts peptides as donor molecules and ligates them to the N-termini of acceptor proteins. Apple ACCO1 has significant homology with RD21 and LeCp. Schaefer *et al.* (2005), in a proteome analysis study employing two-dimensional PAGE and mass spectrometry, observed transpeptidation where two peptides originating in different regions of the αA-crystallin protein were assembled. Peptide ligase activity of ACCO may account for some of the ACCO fragments larger than 15 kDa observed in fragmentation studies (Barlow *et al.* 1997; Zhang *et al.* 1997). In addition to having homology with cysteine proteases, our examination of the ACCO amino acid sequence revealed numerous sequence motifs (Aitken 1999) for putative protein–protein interactions, serine/threonine kinases, tyrosine kinases and glycosylation (Table 3).

### Protein–protein interactions and phosphorylation studies with ACCO

**Figure 15.**
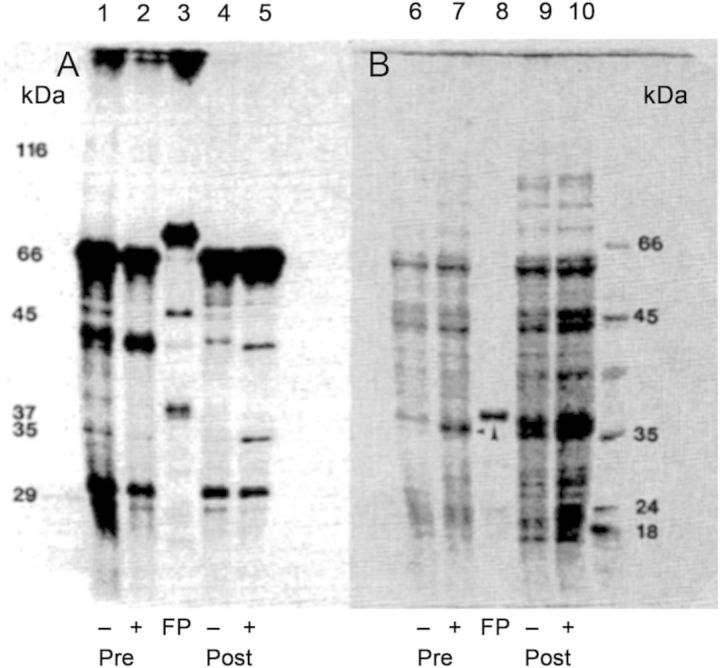
*In vitro* phosphorylation of pre- and postclimacteric apple fruit proteins and the His-Tag recombinant ACCO fusion protein (FP). A particulate membrane fraction from pre- or postclimacteric apple fruit proteins was incubated in [γ-^32^P]ATP in an *in vitro* phosphorylation assay for 30 min in the absence (−) or presence (+) of the ACCO fusion protein or with ACCO alone. The incubation mixtures were then passed through a Ni^2+^ affinity column with the His-Tag ACCO fusion protein attached to bind any proteins in the phosphorylation assay extracts capable of binding to ACCO through protein–protein interactions. The affinity column was then eluted with imidazole to remove ACCO with any bound proteins and subjected to SDS–PAGE. SDS–PAGE would release proteins bound to ACCO. (A) Autoradiogram of SDS–PAGE separation of proteins exposed to the X-ray film. (B) Coomassie Blue-stained proteins from the same SDS–PAGE gel slab. Pre andpost refer to preclimacteric and postclimacteric apple fruit, respectively. Minus and plus signs signify assays conducted without or with the ACCO His-Tag fusion protein, respectively. FP is assay with ACCO alone. The small arrowheads in the FP lane of (B) indicate the position of the fusion protein and a proteolytically cleaved product of the FP in lanes 7 and 8. (From Dilley *et al.* 1995. *Acta Horticulturae* **379**:25–39, with permission from *Acta Horticulturae*.)

Recombinant apple ACCO1 with a His-Tag bound to a Ni^2+^ column was employed as a protein affinity column as ‘bait’ to determine whether any proteins from apple fruit can bind to ACCO through protein–protein interactions (Dilley *et al.* 1995). Products of an *in vitro* phosphorylation assay conducted with a 100 000 *g* membrane fraction collected from pre- and postclimacteric apple fruits in the presence or absence of ACCO were passed through the ACCO affinity column and subjected to SDS–PAGE analysis. A similar assay was done with ACCO only. Numerous proteins of unknown nature from both pre- and postclimacteric apple fruits became differentially phosphorylated (Fig. 15A). Moreover, ACCO became phosphorylated when assayed separately. Protein phosphorylation was more evident in post-climacteric as compared with preclimacteric fruit. A protein of ∼35 kDa became more heavily phosphorylated in post-climacteric as compared with preclimacteric fruit in the presence of ACCO. This 35-kDa protein is not a breakdown product of ACCO because it is absent in lane 3 (Fig. 15A). A strongly phosphorylated protein of ∼70 kDa was found when ACCO was assayed separately. This protein was not detectable by Coomassie Blue staining. The absence of a radiolabel at the mass of ACCO in panel A, lanes 2 and 5, may be due to phosphatase activity in the membrane fraction. Coomassie Blue staining revealed enhanced levels of proteins as apple fruits ripen and this has been known for more than 50 years. Differential phosphorylation of numerous proteins in pre- and postclimacteric apple fruit observed in this *in vitro* study conducted in the presence of recombinant ACCO indicates post-translational modifications not directly related to its enzyme activity in ethylene biosynthesis. The pattern of ^32^P labelling differences between pre- and postclimacteric fruit suggests maturity-dependent changes in kinase and phosphatase activities. Moreover, some of these activities may relate to a role of the ACCO protein (or parts thereof) in ethylene-related signal transduction. The ACCO protein sequence reveals that ACCO has amino acid sequence domains for a putative leucine zipper, SH2/SH3 protein interactions, and phosphokinase and cysteine protease interactions (Table 3) that may account for the protein binding and phosphorylation study results (Fig. 15).

## Conclusions

Much insight has been gained about the ACCO reaction mechanism from X-ray crystallography structure interpretation, spectrophotometric studies, site-directed mutagenesis and steady-state kinetic investigations, DFT analyses and protein modelling studies. Collectively, these studies reveal the nature of the ACCO reaction site and how specific amino acid residues may participate in the catalytic reaction. So far, it appears that the ‘active structure’ of ACCO is formed when ACC becomes bound to the 2-His–1-Asp Fe(II) site allowing the C-terminal α-helix 11 to fold into position near the Fe(II) site. This provides a ‘nest’ comprised of several positively charged amino acid residues from the C-terminal helix along with Lys199 from β-strand 7 and Lys158 from β-strand 5, which serve as binding sites via H-bonds and electrostatic interactions for Asc and bicarbonate to coordinately activate the ACCO reaction. Iron is the binding site for ACC and oxygen. The H-bonding and electrostatic binding sites for ACC, bicarbonate and Asc for *M. domestica* ACCO1 include Arg175, Arg244, Ser246, Lys158, Lys292, Arg299 and Phe300. Arg175 is the most important amino acid affecting ACCO activity. Its role may be structural or it may be a binding site for bicarbonate. Lys292 is the most important Asc binding site followed by Lys158. We propose that Asc may form a Schiff base with one of the two essential lysyl residues. This may serve in the non-redox role of Asc as the base catalyst to activate the reaction but not as the reductant to reduce oxygen. An Asc–Lys Schiff base may play a role in protecting essential lysyl residues more generally in other proteins and enzymes from oxidative damage. Pyridoxal-5′-phosphate would readily replace Asc bound to a lysyl residue as a Schiff base. This concept is worthy of further study. A novel result of our investigation is that cyanide, a product of the ACCO reaction, may become a ligand of ACCO/Fe(II) at the completion of reaction turnover. We propose that cyanide becomes displaced when ACC, Asc and oxygen bind in sequence. Our finding that cyanide protects ACCO from inactivation and also activates the reaction warrants further investigation. Final proof of where and how ACC, Asc and bicarbonate bind to ACCO requires X-ray crystallography analyses of ACCO with substrates and cofactors included along with cyanide serving as an oxygen surrogate. An ACCO/Fe(II)/ACC/CN complex would lend itself to spectrophotometric studies. Such analyses may be possible by employing a closely similar but non-reactive substrate analogue as done by Orville *et al.* (1997) with PCD. Alternately, ACCO mutant Arg299Glu, which has no detectable activity, may be employed for X-ray crystallography analysis with substrates and cofactors. Our resource of ACCO mutant constructs to produce recombinant proteins can be made available to other investigators. We propose that ACCO may play other important roles in the ethylene signal transduction pathway not directly linked to the ACCO reaction. The ACCO protein appears to have post-translational activities including phosphokinase activity, protein–protein interaction capability, and cysteine protease and transpeptidase (peptide ligase) activities. ACC oxidase has significant homology with *L. esculentum* cysteine protease LeCp, which functions as a protease and, after post-translational processing, as a regulator of *Acs2* gene expression (Matarasso *et al.* 2005). ACC oxidase may play a similar role in signal transduction after post-translational processing. *In vitro* studies reveal that ACCO binds to and promotes phosphorylation of apple fruit proteins (of unknown nature) in a ripening-dependent manner. It also becomes autophosphorylated. ACC oxidase becomes inactivated by fragmentation (Barlow *et al.* 1997; Zhang *et al.* 1997) and has intrinsic protease and transpeptidase (ligase) activity. The post-translational activities of ACCO warrant further research.

## Sources of Funding

The study was supported by the United States Department of Agriculture-National Research Initiatives Grant 9602627, National Science Foundation grant to Neogen Corporation, Lansing, Michigan, and the Michigan Agricultural Experiment Station.

## Contributions by the Authors

Z.W. transformed the recombinant and native ACCO proteins in *E. coli*, purified the enzymes and conducted the enzyme assays. D.K.K.-K. prepared ACCO mutants by site-directed mutagenesis. F.V. conducted enzyme purifications, lysine-specific reagent studies, enzyme assays and the cyanide activation studies. R.B. provided analytical assistance and data logistics for manuscript preparation. K.P. assisted in analysis of X-ray structure interpretation of ACCO mutants. D.R.D. designed the experiments, interpreted data and wrote the manuscript.

## Conflict of Interest Statement

None declared.

## Supplementary Material

Additional Information

## Supporting Information

The following Supporting Information is available in the online version of this article –

**Supporting Information Fig. 1.** Sequence comparison of ACC oxidase (ACCO) from *Petunia hybrida* (PDB 1WA6), anthocyanin synthase (PDB 1GP6) and isopenicillin N synthase (PDB 1BK0). The three enzymes are members of the 2-His–1-Asp non-haem iron enzymes. From: Zhang *et al.* (2004). *Chemistry and Biology* 11:1383–1394 (with permission of *Chemistry and Biology*).

**Supporting Information Fig. 2.** Lysine-specific reagent AMCA-sulfo-NHS inhibition up to a 2/1 mol/mol ratio indicates two essential lysine residues in ACCO. Purified recombinant ACCO (0.8 nmol) was treated with AMCA-sulfo-NHS for 10 min in MOPS buffer (pH 7.2) and the reaction was stopped with 50 µM glycine. ACC oxidase assay cofactors were added and ACC was used to start the assay. Sulfo-NHS blocks the ε-amino of lysine.

**Supporting Information Fig. 3.** Pyridoxal-5′-phosphate (PLP) competitively inhibits ACCO with respect to Asc. Double reciprocal plot of ACCO activity versus Asc concentration. One nmole of purified ACCO fusion protein was assayed with the standard assay with 0 µM (circles), 100 µM (squares), 200 µM (triangles) and 300 µM (diamonds) PLP with Asc varied.

**Supporting Information Fig. 4.** d-Saccharic acid 1,4-lactone (a structural analogue of Asc) is a competitive inhibitor of ACCO with respect to Asc (*K*_i_ = 0.3 mM). It apparently binds to the high-affinity Asc binding site as shown by Rocklin *et al.* (2004). The purified ACCO fusion protein was assayed with the standard assay (see Methods) with 0 mM (circles), 0.5 mM (squares) and 1.5 mM d-saccharic 1,4-lactone.

**Supporting Information Fig. 5.** Double reciprocal plot of ACCO activity vs. Asc concentration with the indicated concentrations of 2,4-pteridinediol (lumazine) showing competitive activation of ACCO. The 2,4-pteridinediol *K*_a_ = 2.2 mM, which is similar to the Asc *K*_a_ of 2.58 mM. 2,4-Pteridinediol activates ACCO in the absence of Asc. Purified recombinant ACCO (1 nmol) was assayed with the standard assay.

**Supporting Information Fig. 6.** 2,4,5-Triamino-6-hydroxypyrimidine (TAP) inhibits ACCO competitively with respect to Asc. Purified recombinant ACCO (1 nmol) was assayed with the standard assay. Inhibition was not reversed at higher ferrous sulfate levels. 4,5,6-Triaminopyrimidine uncompetitively activates ACCO with respect to Asc (data not shown).
