# Supplementary material for: 1-Aminocyclopropane-1-carboxylic acid oxidase reaction mechanism and putative post-translational activities of the ACCO protein
Source: AoB Plants. 2013 Aug 1;5:plt031. doi: 10.1093/aobpla/plt031 (PMC3828642; doi:10.1093/aobpla/plt031)
Supplement: Additional Information [file supp_5_plt031_index.html]

1-Aminocyclopropane-1-carboxylic acid oxidase reaction mechanism and putative post-translational activities of the ACCO protein — Additional Information 

# 1-Aminocyclopropane-1-carboxylic acid oxidase reaction mechanism and putative post-translational activities of the ACCO protein

## Additional Information

**Files in this Data Supplement:**

- Additional Information - pdf file
