# Supplementary material for: 1-Aminocyclopropane-1-carboxylic acid oxidase reaction mechanism and putative post-translational activities of the ACCO protein
Source: AoB Plants. 2013 Aug 1;5:plt031. doi: 10.1093/aobpla/plt031 (PMC3828642; doi:10.1093/aobpla/plt031)
Supplement: Additional Information [file supp_plt031_plt031supp.pdf]

## Supporting Information Legends

**Supporting Information Fig. 1.**

Sequence comparison of ACCO from *Petunia hybrida* (PDB 1WA6), anthocyanin synthase (PDB 1GP6) and isopenicillin N synthase(PDB 1BK0). The three enzymes are members of the 2-His-1-Asp non-heme iron enzymes. From: Zhang *et al.* 2004. *Chemistry and Biology* 11:1383-1394 (with permission of Chemistry and Biology)..

**Supporting Information Fig. 2.**

Lysine-specific reagent AMCA-sulfo-NHS inhibition up to 2/1 mol/mol ratio indicates 2 essential lysine residues in ACCO. Purified recombinant ACCO (0.8 nmol) was treated with AMCA-sulfo-NHS for 10 minutes in MOPS buffer (pH 7.2) and the reaction was stopped with 50 µM glycine. ACCO assay cofactors were added and ACC was used to start the assay. Sulfo-NHS blocks the €-amine of lysine.

**Supporting Information Fig.3.**

Pyridoxal-5'-phosphate(PLP) competitively inhibits ACCO with respect to ascorbate. Double reciprocal plot of ACCO activity versus ascorbate concentration. One nmol of purified ACCO fusion protein was assayed with the standard assay with 0 (circles),100 (squares), 200 (triangles) and 300 (diamonds) µM PLP with ascorbate varied.

**Supporting Information Fig.4.**

D-Saccharic acid 1,4-lactone (a structural analog of ascorbic acid) is a competitive inhibitor of ACCO with respect to ascorbic acid ($K_i$ = 0.3 mM). It apparently binds to the high affinity ascorbate binding site as shown by Rocklin *et al.* (2004). The purified ACCO fusion protein was assayed with the standard assay (see Methods) with 0 mM (circles), 0.5 mM (squares) and 1.5 mM D-saccharic 1,4-lactone.

**Supporting Information Fig. 5.**

Double reciprocal plot of ACCO activity vs ascorbic acid concentration with the indicated concentrations of 2,4-pteridinediol (lumazine) showing competitive activation of ACCO. The 2,4-pteridinediol $K_{activation}$ = 2.2 mM. which is similar to the ascorbic acid $K_a$ of 2.58 mM. 2.4-Pteridinediol activates ACCO in the absence of ascorbic acid. Purified recombinant ACCO (1 nmol) was assayed with standard assay.

**Supporting Information Fig. 6.**

2,4,5-Triamino-6-hydroxypyrimidine (TAP) inhibits ACCO competitively with respect to ascorbic acid. Purified recombinant ACCO (1 nmol) was assayed with the standard assay. Inhibition was not reversed at higher ferrous sulfate levels. 4,5,6-Triaminopyrimidine uncompetitively activates ACCO with respect to ascorbic acid (data not shown).

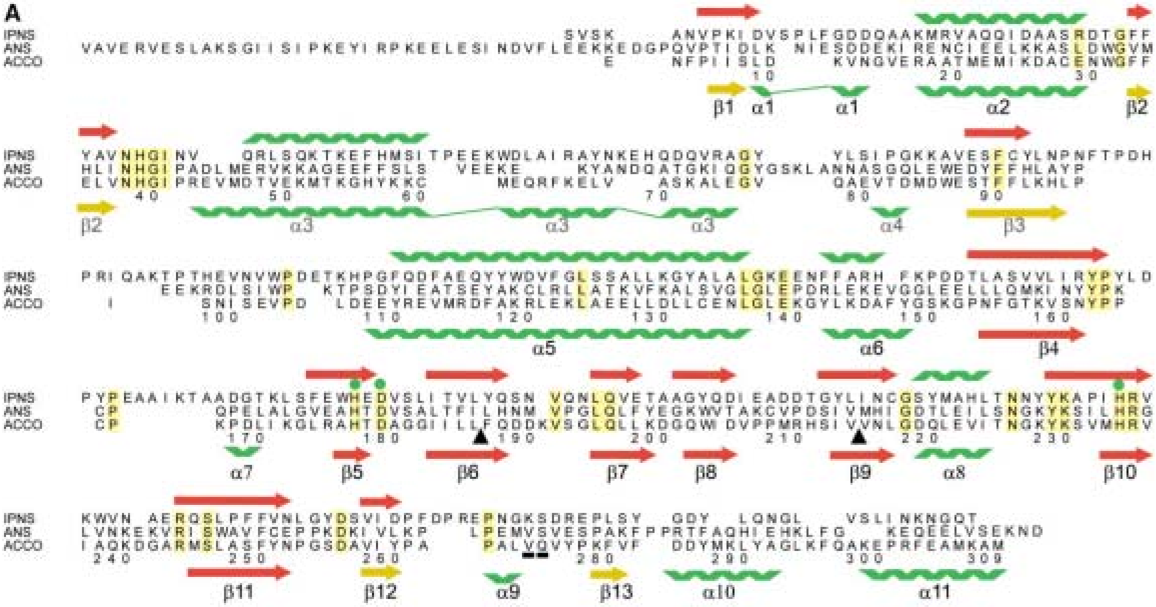
A
IPNS SVSK ANVPKI DVSPLFGDDQAAKMRVAQQIDAASRDTGFF
ANS VAVERVESLAKSGIISIPKEYIRPKEELESINDVFLEEKKEDGPQVPTIDLK NIESDDEKIRENCIEELKKASLDWGVM
ACCO E NFPIISLD KVNGVERAATMEMIKDACENWGFF
10 20 30
β1 α1 α1 α2 β2
IPNS YAVNHGINV QRLSQKTKEFHMSITPEEKWDLAIRAYNKEHQDQVRAGY YLSIPGKKAVESFCYLNPNFTPDH
ANS HLINHGIPADLMERVKKAGEEFFSLS VEEKE KYANDQATGKIQGYGSKLANNASGQLEWEDYFFHLAYP
ACCO ELVNHGIPREVMDTVEKMTKGHYKKC MEQRFKELV ASKALEGV QAEVTDMDWESTFFLKHLP
40 50 60 70 80 90
β2 α3 α3 α3 α4 β3
IPNS PRIQAKTPTHEVNVWPDETKHPGFQDFAEQYYWDVFGLSSALLKGYALALGKEENFFARH FKPDDTLASVVLIRYPYLD
ANS EEKRDLSIWP KTPSDYIEATSEYAKCLRLLATKVFKALSVGLGLEPDRLEKEVGGLEELLLQMKINYYPK
ACCO I SNISEVPD LDEEYREVMRDFAKRLEKLAEELLDLLCENLGLEKGYLKDAFYGSKGPNFGTKVSNYPP
100 110 120 130 140 150 160
α5 α6 β4
IPNS PYPEAAIKTAADGTKLSFEWHEDVSLITVLYQSN VQNLQVETAAGYQDIEADDTGYLINCGSYMAHLTNNYYKAPIHRV
ANS CP QPELALGVEAHTDVSALTFILHNM VPGLQLFYEGKWVTAKCVPDSIVMHIGDTLEILSNGKYKSILHRG
ACCO CP KPDLIKGLRAHTDAGGIILLFQDDKVSGLQLLKDGQWIDVPPMRHSIVVNLGDQLEVITNGKYKSVMHRV
170 180 190 200 210 220 230
α7 β5 β6 β7 β8 β9 α8 β10
IPNS KWVN AERQSLPFFVNLGYDSVIDPFDPREPNGKSDREPLSY GDY LQNGL VSLINKNGQT
ANS LVNKEKVRISWAVFCEPPKDKIVLKP LPEMVSVESPAKFPPRTFAQHIEHKLFG KEQEELVSEKND
ACCO IAQKDGARMSLASFYNPGSDAVIYPA PALVQVYPKFVF DDYMKLYAGLKFQAKEPRFEAMKAM
240 250 260 280 290 300 309
β11 β12 α9 β13 α10 α11

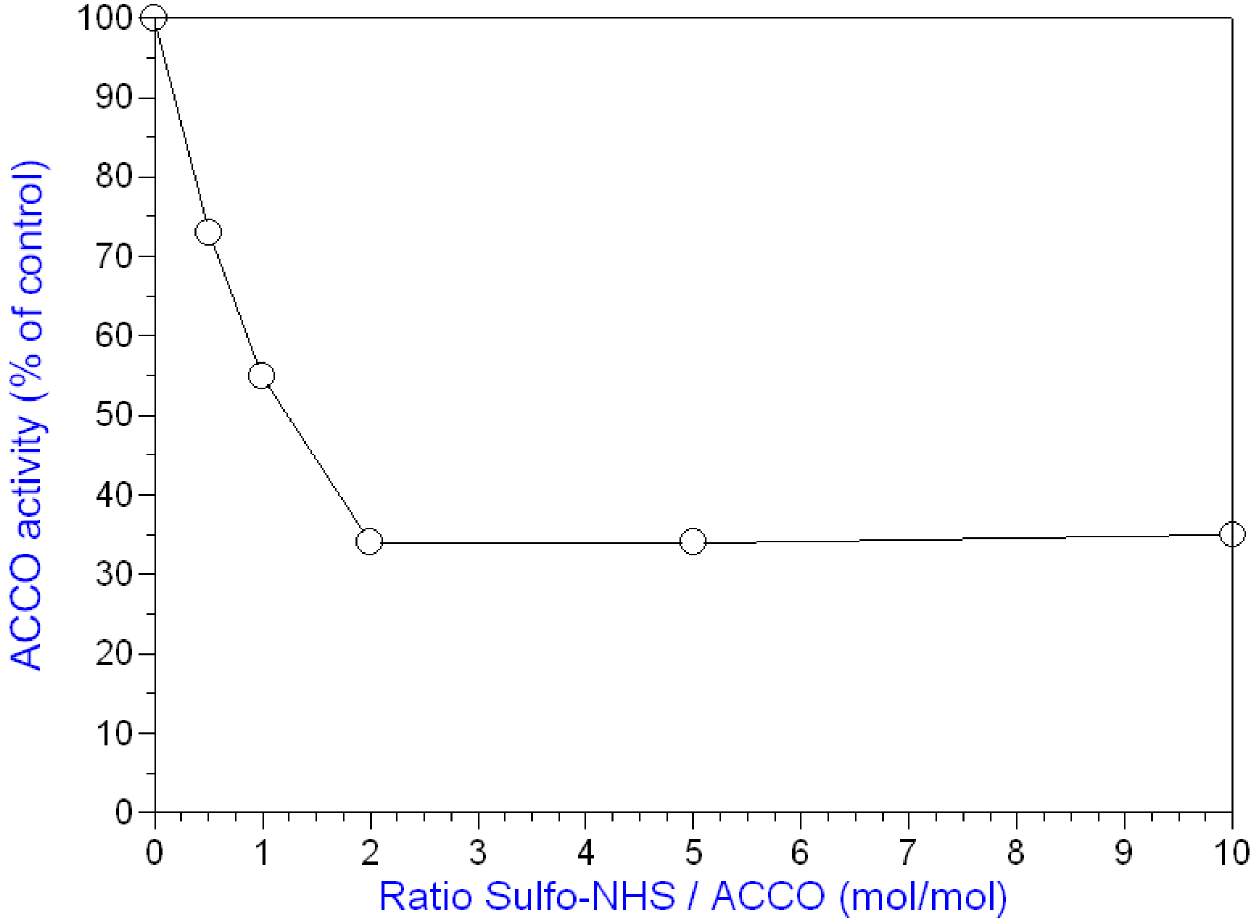
ACCO activity (% of control)
Ratio Sulfo-NHS / ACCO (mol/mol)
100
90
80
70
60
50
40
30
20
10
0
0
1
2
3
4
5
6
7
8
9
10

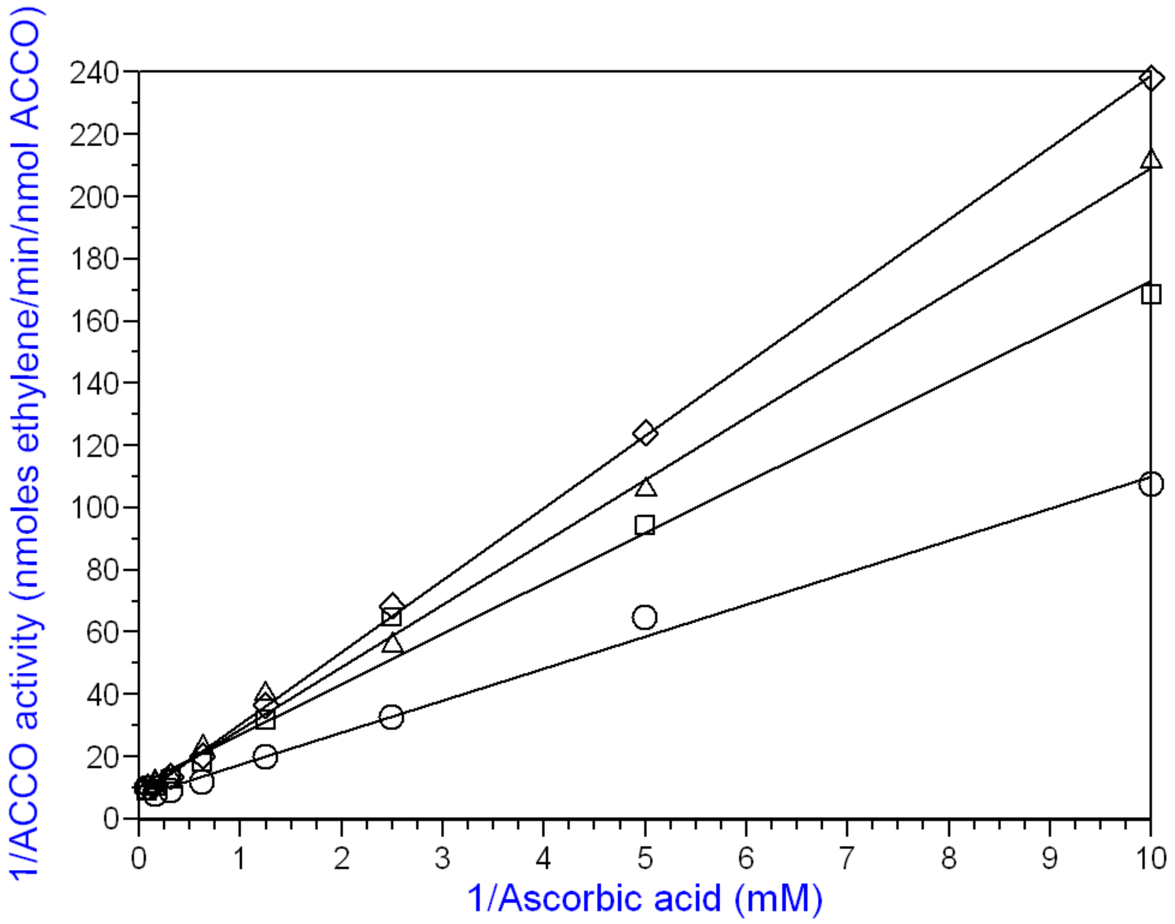

1/ACCO activity (nmoles ethylene/min/nmol ACCO)
240
220
200
180
160
140
120
100
80
60
40
20
0
0
1
2
3
4
5
6
7
8
9
10
1/Ascorbic acid (mM)

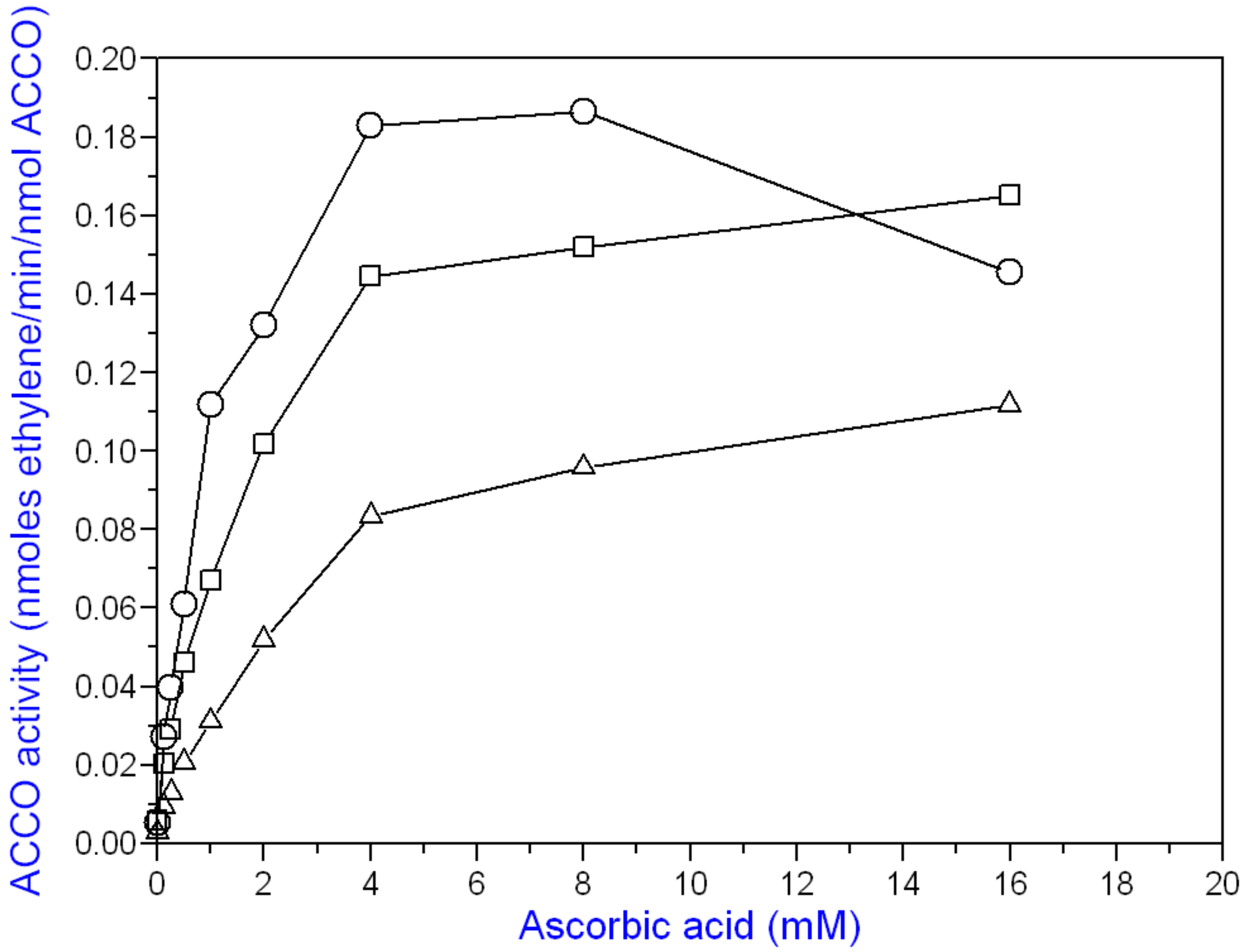
ACCO activity (nmoles ethylene/min/nmol ACCO)
0.00
0.02
0.04
0.06
0.08
0.10
0.12
0.14
0.16
0.18
0.20
0
2
4
6
8
10
12
14
16
18
20
Ascorbic acid (mM)

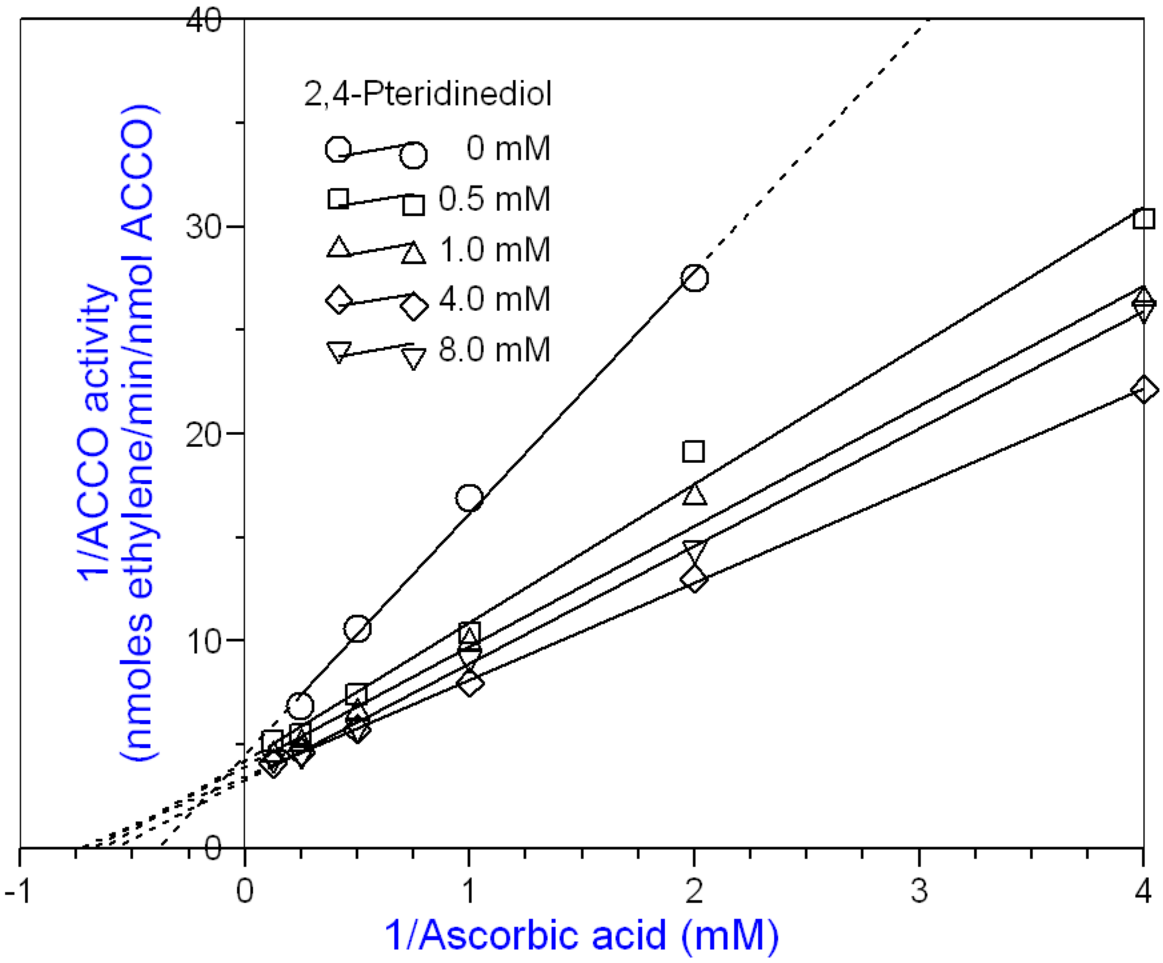

2,4-Pteridinediol
0 mM
0.5 mM
1.0 mM
4.0 mM
8.0 mM
1/ACCO activity
(nmoles ethylene/min/nmol ACCO)
1/Ascorbic acid (mM)
0
10
20
30
40
-1
0
1
2
3
4

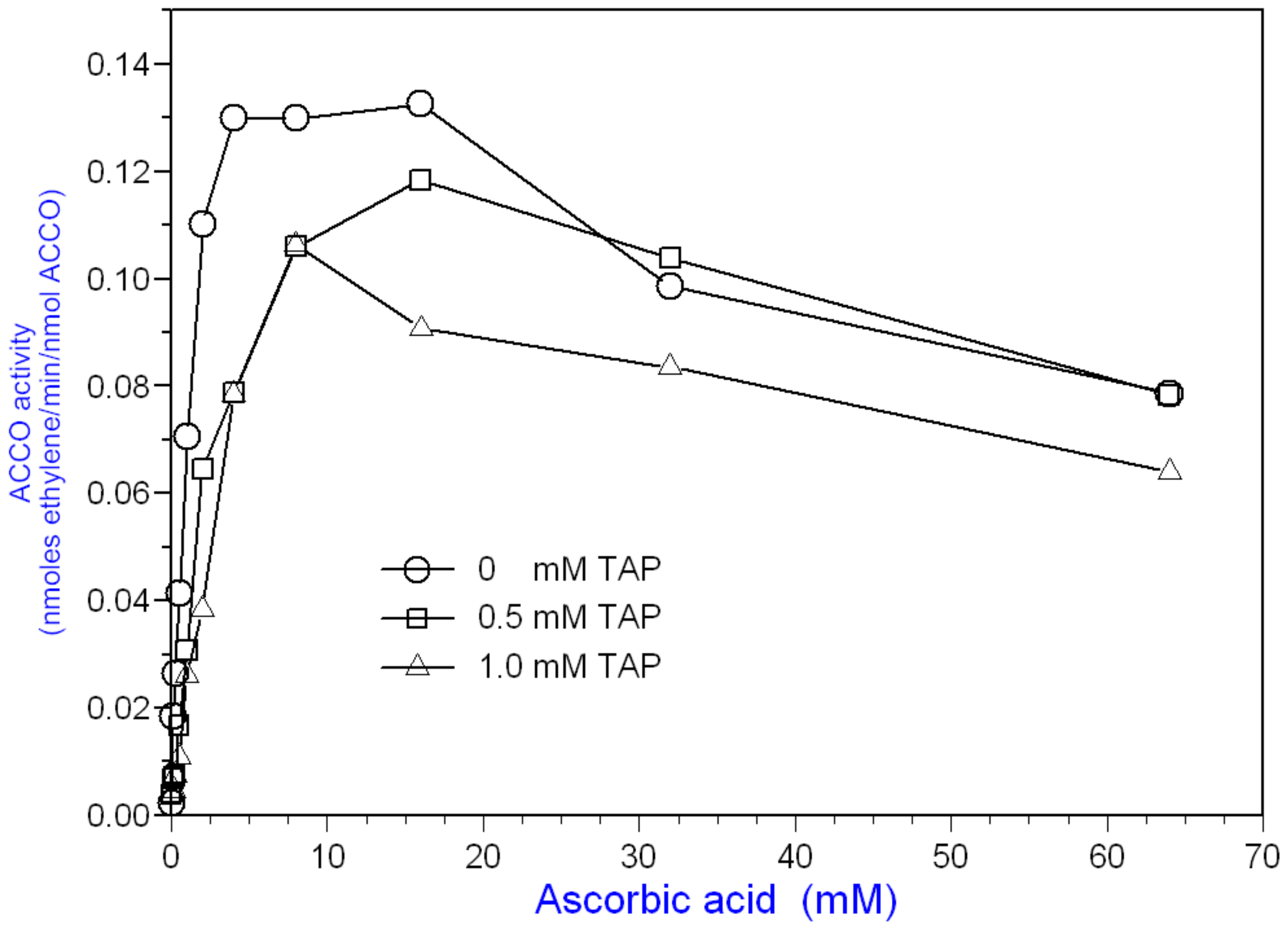
ACCO activity
(nmoles ethylene/min/nmol ACCO)
0.00
0.02
0.04
0.06
0.08
0.10
0.12
0.14
0
10
20
30
40
50
60
70
Ascorbic acid (mM)
0 mM TAP
0.5 mM TAP
1.0 mM TAP
